# Optimising cluster survey design for planning schistosomiasis preventive chemotherapy

**DOI:** 10.1371/journal.pntd.0005599

**Published:** 2017-05-26

**Authors:** Sarah C. L. Knowles, Hugh J. W. Sturrock, Hugo Turner, Jane M. Whitton, Charlotte M. Gower, Samuel Jemu, Anna E. Phillips, Aboulaye Meite, Brent Thomas, Karsor Kollie, Catherine Thomas, Maria P. Rebollo, Ben Styles, Michelle Clements, Alan Fenwick, Wendy E. Harrison, Fiona M. Fleming

**Affiliations:** 1 Schistosomiasis Control Initiative, Department of Infectious Disease Epidemiology, Imperial College London, St. Mary’s Campus, Norfolk Place, London, United Kingdom; 2 The Royal Veterinary College, Hawkshead Lane, Hatfield, Hertfordshire, United Kingdom; 3 London Centre for Neglected Tropical Disease Research, London, United Kingdom; 4 Global Health Group, University of California San Francisco, San Francisco, California, United States of America; 5 Department of Infectious Disease Epidemiology, Imperial College London, St. Mary’s Campus, Norfolk Place, London, United Kingdom; 6 Ministry of Health, Capital City, Lilongwe 3, Malawi; 7 Ministry of Health and Social Welfare of Côte d’Ivoire, Abidjan, Côte d’Ivoire; 8 Fliarial Programme Support Unit, Liverpool School of Tropical Medicine, Pembroke Place, Liverpool, United Kingdom; 9 Neglected Tropical and Non Communicable Diseases Program, Ministry of Health and Social Welfare, Monrovia 10, Liberia; 10 National Foundation for Educational Research, Upton Park, Slough, United Kingdom; George Washington University, UNITED STATES

## Abstract

**Background:**

The cornerstone of current schistosomiasis control programmes is delivery of praziquantel to at-risk populations. Such preventive chemotherapy requires accurate information on the geographic distribution of infection, yet the performance of alternative survey designs for estimating prevalence and converting this into treatment decisions has not been thoroughly evaluated.

**Methodology/Principal findings:**

We used baseline schistosomiasis mapping surveys from three countries (Malawi, Côte d’Ivoire and Liberia) to generate spatially realistic gold standard datasets, against which we tested alternative two-stage cluster survey designs. We assessed how sampling different numbers of schools per district (2–20) and children per school (10–50) influences the accuracy of prevalence estimates and treatment class assignment, and we compared survey cost-efficiency using data from Malawi. Due to the focal nature of schistosomiasis, up to 53% simulated surveys involving 2–5 schools per district failed to detect schistosomiasis in low endemicity areas (1–10% prevalence). Increasing the number of schools surveyed per district improved treatment class assignment far more than increasing the number of children sampled per school. For Malawi, surveys of 15 schools per district and 20–30 children per school reliably detected endemic schistosomiasis and maximised cost-efficiency. In sensitivity analyses where treatment costs and the country considered were varied, optimal survey size was remarkably consistent, with cost-efficiency maximised at 15–20 schools per district.

**Conclusions/Significance:**

Among two-stage cluster surveys for schistosomiasis, our simulations indicated that surveying 15–20 schools per district and 20–30 children per school optimised cost-efficiency and minimised the risk of under-treatment, with surveys involving more schools of greater cost-efficiency as treatment costs rose.

## Author summary

Many countries are currently scaling up efforts to control schistosomiasis, a helminthic disease for which preventive chemotherapy with praziquantel is the main control tool. In order to apply WHO guidelines on how frequently to treat a given district or similar geographic unit for schistosomiasis, survey-based estimates of infection prevalence are required. However, the optimal size and design of survey for generating such data is not clear, and there is a clear trade-off between accuracy and cost–larger surveys provide more accurate information with which to target treatment, but cost more to carry out. Here, we systematically assess what size and design of simple 2-stage cluster survey (where primary school children are tested for infection), might best enable control programmes to implement WHO treatment guidelines. We use empirical data on schistosomiasis distribution from three African countries together with computer simulations to compare survey performance, in terms of accuracy and cost-efficiency–the ability of a survey to accurately determine treatment frequency, per unit cost. We show that although small surveys of around 5 schools per district are frequently adopted for mapping schistosomiasis, such small surveys are prone to miss endemic schistosomiasis fairly often, and are also not cost efficient. Our results suggest that among the designs tested, surveys involving 15–20 schools per district optimise cost-efficiency, providing the most accurate treatment decisions per dollar spent. These findings have important implications for the schistosomiasis control community, and provide the first evidence-based suggestion of a simple survey design for mapping schistosomiasis in endemic countries.

## Introduction

Schistosomiasis is a major global health problem, and is estimated to infect 230 million people, cause at least 11,000 deaths per year [1,2] and 3.3 million Disability Adjusted Life Years in 2010 [3]. While various tools are used to control the disease, population-level preventive chemotherapy (PC) with praziquantel is currently the cornerstone of schistosomiasis control, and PC-based programmes are scaling up across Africa [4,5].

Since schistosomiasis shows high spatial heterogeneity in its geographic distribution, and school-age children (SAC, aged 5–14 years) are an important high-risk group [6], PC is targeted to those areas at greatest risk of infection and focuses on this age group. Treating all SAC in such areas remains more cost-effective than individual-level test-and-treat strategies [7,8] and is achieved by assigning geographic areas to WHO-recommended treatment classes based on infection prevalence (Table 1) [6,9]. Thus, to decide treatment classes, epidemiological data must first be collected to ascertain prevalence. Published WHO guidelines do not provide specific guidance on survey design for this purpose, but recommend collecting prevalence data according to ‘ecological zones’ [6]. Ecological zones are not well defined, however, and it is unclear how to convert ecological zone-based prevalence data into treatment decisions, which are commonly applied across implementation units, such as health or educational districts. As a result, a variety of survey designs have been adopted [10–14] and the performance of alternative designs has not been thoroughly evaluated.

**Table 1:** WHO treatment guidelines for schistosomiasis, according to estimated prevalence. Adapted from [6,9].

Endemicity level	Schistosomiasis prevalence (pooled species) based on parasitological methods	WHO recommended treatment strategy
Non-endemic	<1%	No treatment
Low	≥1% and <10%	Treat school-age children twice during primary school years
Moderate	≥10 and <50%	Biennial treatment of all school-age children; as well as special risk groups in adults
High	≥50%	Annual treatment of all school-age children; as well as special risk groups in adults

Computerized sampling simulations have proved useful for assessing alternative survey approaches for various tropical diseases, including soil-transmitted helminthiases, trachoma and intestinal schistosomiasis [15–17]. These entail generating ‘‘gold standard” datasets that maintain the spatial heterogeneity observed in empirical datasets, and then comparing how well alternative sampling approaches perform in estimating known parameters from this spatially realistic simulated data. Such simulations allow multiple approaches to be tested with far greater replication than would be possible in field tests. A previous simulation study on schistosomiasis [15] compared the performance of two methods to estimate school level infection prevalence, lot quality assurance sampling (LQAS) and a spatial grid-based survey combined with spatial interpolation. Here, we focus on comparing simple two-stage cluster survey designs that aim to estimate schistosomiasis prevalence among SAC for a set of implementation units, such as districts. This simple survey design is often used when more complex (e.g. spatially informed) survey designs are not possible, when the spatial distribution of infection within districts is thought to be relatively homogeneous, or when a simple, easy to analyse survey is desired. For example, the design of spatially stratified surveys requires reliable information on the likely distribution of disease, as well as accurate location data for all potential sampling locations. Such information may not available for the entire area to be surveyed. Two-stage cluster surveys involving a simple random sample of sites from a list in each implementation unit, have therefore been used to generate implementable PC plans in a number of countries [18–21]. We apply sampling simulations to data from three African countries in order to assess what number of sampling sites, and number of individuals screened at each site, maximises survey accuracy and cost-efficiency respectively. Survey accuracy is expected to increase asymptotically with survey size, with diminishing returns as surveys get larger. However, a curved relationship between survey size and cost-efficiency is expected, with the optimum reflecting a balance between survey accuracy and cost. Cost-efficiency should increase with survey size initially, but then decrease as the costs of very large surveys outweigh their accuracy benefits. The key question then, is at what point this curve turns, i.e. what survey size leads to the most accurate treatment decisions per unit cost?

## Methods

### Ethics statement

Ethical approval for the surveys analysed here (including the consent process and all methodology) was obtained from Imperial College London Research Ethics Committee (ICREC_8_2_2). Surveys were performed as part of national schistosomiasis and soil transmitted helminth control programmes in all countries, overseen and approved by the Ministry of Health. In Malawi, all participants provided written consent. In Côte d’Ivoire and Liberia, as participants were under the age of 18 years the Ministry of Health required written consent be provided by adults. As literacy levels were very low, written consent from every parent or guardian was not possible, and each head teacher provided written consent for the survey and all individual participants provided oral consent. In Liberia, headteachers also received oral consent from parents at Parent Teacher Association (PTA) meetings before surveys began, with parent presence at PTA meetings documented on an attendance register. Only children who consented either in writing (in Malawi) or orally (in Côte d’Ivoire and Liberia) took part in the surveys.

### Characterising spatial heterogeneity in schistosomiasis from empirical datasets

**Fig 1:**
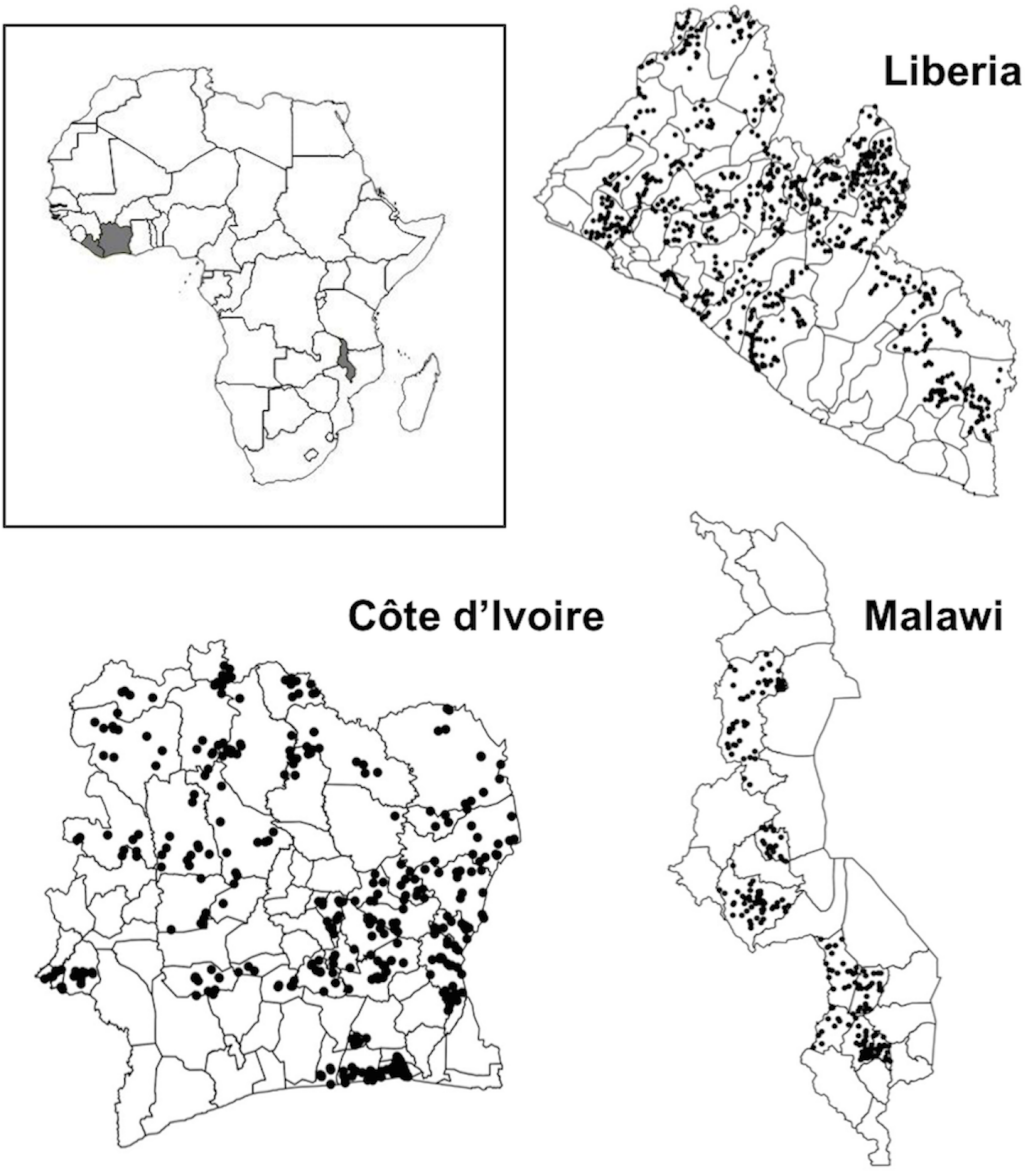
Spatial distribution of geo-referenced primary schools involved in schistosomiasis baseline mapping surveys in Malawi (n = 244), Côte d’Ivoire (n = 500) and Liberia (n = 933). Data from these sites were used to estimate semivariograms and generate spatially realistic gold standard datasets on which sampling simulations were performed.

All analyses were performed in R v3.1.0. Data from baseline mapping surveys in Malawi, Côte d’Ivoire and Liberia were used to characterise spatial heterogeneity in schistosomiasis prevalence (Table 2; Fig 1). All surveys adopted the same two-stage cluster survey protocol. This involved randomly selecting 15–20 primary schools in each implementation unit (health or educational district), then selecting 30 children per school (with an even gender ratio) to be tested for *Schistosoma haematobium* using a single urine filtration and *S*. *mansoni* using duplicate Kato-Katz slides from a single stool. At each school, children aged 10–14 were eligible for participation. Where age was difficult to determine, grades corresponding to the targeted age group were used. Children were selected systematically at each school, by assembling a line each of eligible boys and girls, and using a sampling interval to select the required 15 children of each gender. Survey sample size was originally decided based on precision-based sample size calculations (S1 Text). We used semivariograms to characterise spatial heterogeneity in the prevalence of schistosomiasis (infection by either *S*. *haematobium*, *S*. *mansoni*, or either species), in each country. Before creating semivariograms, logistic regression models were performed to remove large-scale spatial trends in prevalence. For each country, individual level infection (1/0) was modelled as a function of longitude and latitude, with school as a random factor, using a binomial mixed model in the R package *lme4*. Where either longitude or latitude did not explain significant variation in prevalence (i.e. p>0.05 in likelihood ratio tests), these terms were removed from the final model. Using the R package *geoR*, omnidirectional semivariograms were then fitted for each country to the school level random effects, with weighted least squares fits of exponential, spherical, and Gaussian models. To further characterise spatial heterogeneity in schistosomiasis prevalence and provide parameters useful in future sample size calculations, we calculated the intra-cluster correlation coefficient (ICC) for schistosomiasis prevalence using the *iccbin* function in R package *aod* [22]. The ICC is a measure of the relatedness or similarity of clustered data, with values ranging from 0 to 1. As the ICC increases, the more individuals within clusters, and the less individuals in different clusters, resemble one another. In the context of our surveys, the higher the district-level ICC, the more similar children in the same school are in their infection status, compared to children in different schools. In sample size calculations, ICC determines the design effect; higher ICC values increase the design effect and the sample size required to obtain a given level of precision for parameter estimates such as prevalence.

**Table 2:** Characteristics of schistosomiasis surveys used to general theoretical gold standard datasets.

Country	Survey date	Mapping units	Districts mapped (N districts in country)	Median size of districts nationally (range)	N geo-referenced schools	Schistosomiasis prevalence (%)	*S*. *haematobium* prevalence (%)	*S*. *mansoni* prevalence (%)
Malawi	February 2012	13	8 (26)	3,194km^2^ (760 to 11,405 km^2^)	244	19.4	16	5.4
Côte d’Ivoire	Dec 2013 to March 2014	43	43 (82)	2,732 km^2^ (25 to 19,306km^2^)	500	6.5	2.7	4
Liberia	March/April 2012 & March/April 2013	58	58 (66)	1,266 km^2^ (107 to 4,610 km^2^)	933	25.4	9	21

### Generation of gold standard datasets

Spatially realistic sampling locations were required to create gold standard datasets. Since geo-referenced data on primary school locations was only available for one of the three countries (Malawi), locations were simulated. We used PPS (“probability proportional to size”) sampling according to population density (data from Worldpop, http://www.worldpop.org.uk/) in package *raster* [23] to assign primary schools to locations in each country (Table A in S2 Text). Geo-referenced school datasets from Malawi and Kenya were used to validate this approach. Good correspondence was observed between simulated and actual school locations across both countries, which was improved by assigning schools separately to large cities (population >1m; Lilongwe, Blantyre and Nairobi) and to the rest of the country (Fig A and Fig B in S3 Text).

We focused simulations on survey designs estimating pooled *S*. *haematobium* and *S*. *mansoni* prevalence, since treatment guidelines are based on pooled schistosomiasis prevalence (Table 1). Conditional simulation was performed using semivariogram parameters to generate 1000 different realizations per country that reproduced the global characteristics of the source data. For each country, the semivariogram model for pooled schistosome infection with the lowest sum of squares was chosen as the best fit for conditional simulation. For Malawi and Liberia, conditionally simulated random effect values were added to predicted trend surfaces (predictions using latitude and longitude) on the log odds scale before being back transformed to prevalence. For Côte d’Ivoire, where no spatial autocorrelation in schistosomiasis was detected (and therefore conditional simulation could not be performed) random effect values were assigned to each school by sampling from the distribution of observed school random effect values. To generate a distribution of random effect values to sample from, the distribution of observed random effects was smoothed using kernel smoothing. Overall, we created 1000 different schistosomiasis prevalence realizations for 5,239 primary school locations in Malawi, 11,429 in Côte d’Ivoire and 2,785 in Liberia (Table A in S2 Text). Based on data provided by government ministries or enrolment figures collected during mapping surveys (Table A in S2 Text), we assumed a population of school-age children (SAC, defined by the WHO as children aged 5–14) per school of 400 for Malawi, 250 for Côte d’Ivoire and 150 for Liberia, and thus calculated the number of SAC that were infected and uninfected with schistosomiasis at each school in each realization.

### Testing alternative survey designs

We simulated two-stage cluster surveys involving a random sample of 2, 5, 10, 15 or 20 schools per district, and a random sample of 10, 20, 30, 40 or 50 SAC per school. For each realization, the *survey* package in R [24] was used to calculate district prevalence and 95% confidence intervals with the ‘beta’ method for proportions. Districts were classified into WHO endemicity and associated treatment classes according to point prevalence estimates (Table 1) [6]. We assessed the accuracy of each survey design across 1000 realizations using four key metrics: (1) the proportion of times that treatable levels of infection (≥1% prevalence) were not detected (2) prevalence estimate precision, as reflected by the width of the 95% confidence interval (3) the proportion of times districts were misclassified (assigned to the wrong treatment class) and (4) the proportion of times districts were under-classified (assigned to a treatment class below their true class). We also explored how three alternative district assignment rules performed, wherein districts were placed into the next highest treatment class if their prevalence estimate was within either 2 or 5 percentage points of the 10% or 50% threshold (“boosting rules"), or if the upper 95% confidence limit overlapped a higher treatment class threshold (“upper CL rule”). To ensure comparability across all survey designs, districts with fewer than 20 schools were excluded from analyses. This resulted in each survey design being assessed 1000 times across a total of 143 districts from the three countries (26 districts in Malawi, 78 in Côte d’Ivoire and 39 in Liberia, Table A in S2 Text). We further explored how survey accuracy according to these four metrics varied with district size.

### Cost analysis

The cost of each survey design was estimated for Malawi using an ingredients based approach [25], using itemized cost data from a 2014 schistosomiasis/STH mapping survey conducted in Malawi. We assumed each survey covered the whole country and monitored *S*. *haematobium* and *S*. *mansoni*. Only financial costs were estimated, with some costs fixed (invariant of sampling strategy) and others variable (depending on survey design; Table 3). Capital item costs were annualized over their useful lifespan (Table B in S2 Text) and a wastage factor of 10% was applied to all relevant items [26]. Based on field experience in Malawi, we assumed three survey teams of constant size worked in parallel, and that survey duration (and hence staff salary costs) changed with survey design (Table C in S2 Text). Survey design-specific travel distances for fuel costs were estimated in qGIS: for each district and for each number of schools, a single random selection of schools was taken from the geo-referenced school database and the shortest path linking them was used as an estimate of average distance travelled per district. All costs were adjusted to US dollars (US$) using the July 1st, 2014 exchange rates of 399.216 Malawian Kwacha (MWK) and 0.586 UK pounds to 1 US dollar (www.oanda.com/convert/classic).

**Table 3:** Itemised costs of school-based surveys for *Schistosoma haematobium* and *S*. *mansoni* in Malawi.

Unit and Cost type	Unit cost§ (2014 prices in USD)	Number required per survey team
Capital equipment* (Fixed)		
Wash basins	4.01	2
Brushes	5.79	2
Buckets	4.01	2
Droppers	0.20	2
Forceps	2.70	4
GPS	123.79	1
Hole punch	14.90	1
Microscope	1,186.97	2
Sieving mesh	37.17	2
Slide boxes	5.99	1
Tally counters	4.13	2
Capital equipment* (Variable)		
Filter Holders	6.51	2 × number of children sampled per school^τ^
Stool pots	0.15	Number of children sampled per school ^τ^
Syringes	0.16	Number of children sampled per school ^τ^
Kato-Katz kit (template and plastic spatula)	0.112	Number of children sampled per school ^τ^
Urine Pots	0.15	Number of children sampled per school ^τ^
**Consumables (Fixed)**		
Marker pens	0.90	2
Notebooks	2.15	5
Pencils	0.38	5
Pens	0.85	5
Scissors	1.81	2
Bleach (500ml)	3.01	1
Insecticide	2.26	1
Soap (400g)	1.24	1
**Consumables (Variable)**		
Methylene blue (25mg)	23.14	0.5 grams per district ^τ^
Glycerol (5L)	31.44	50 ml per district ^τ^
Iodine (10ml)	3.00	1 per district ^τ^
Newspaper	0.25	1 per district
Bin bags	0.15	2 per school sampled ^τ^
Cellophane	0.55	1 sheet per 40 children sampled ^τ^
Filters	0.072	1 for each child sampled^τ^
Folders	1.46	0.5 per school sampled
Gloves	0.05	60 per school sampled ^τ^
Hemastix	0.52	0.5 for each child sampled ^τ^
Masking tape	2.26	0.2 per school sampled ^τ^
Microscope slides†	0.03	3† for each child sampled ^τ^
Stool plastic (100 yards)	10.56	1.5 yards per school sampled ^τ^
Tissue paper	0.67	0.2 per school sampled ^τ^
Washing powder (1kg)	3.16	60 grams per school sampled ^τ^
Photocopying	0.02	1 sheet per child sampled and 3 per school sampled ^τ^
**Salaries (Variable)**		
District health officer	25.00 per day	Days required for survey^¥^ (Table C in S2 Text)
Driver	45.58 per day	Days required for survey^¥^ (Table C in S2 Text)
Supervisor	57.61 per day	Days required for survey^¥^ (Table C in S2 Text)
Technicians (four needed)	50.10 per day	4 × days required for survey^¥^ (Table C in S2 Text)
**Transport**		
Fuel	0.53 per km	The number of schools sampled^‡^ × the average distance (km)# between schools ^#^
Maintenance	166.99	1

* Capital items (resources lasting longer than a year/ can be re-used in subsequent surveys). Shown unit costs have not been annualized to allow comparison with other reported costs (though they were when estimating the total cost of each mapping strategy–see methods).

† Three slides are needed per child sampled, assuming duplicate slides are screened for *S*. *mansoni* and one slide for *S*. *haematobium*.

¥ The number of days required to survey a district under a given sampling design (Table C in S2 Text) × the number of districts surveyed per team (the total number of implementation units (26) divided by the number of teams)

τ +10% wastage

‡ For each district an extra journey worth of fuel was accounted for the trip back from the field to a central point.

# Average distance between schools: 2 schools (39km), 5 schools (32.5km), 10 schools (22km), 15 schools (17.5km), 20 schools (16km).

Per-capita treatment cost was assumed to be $0.30, based on financial costs of programme delivery and purchased praziquantel costs in Malawi for 2014, but was varied from $0.10 to $0.60 in sensitivity analyses [27–29]. The total cost for each design was calculated by summing survey costs and the cost of treating the entire Malawi SAC population (4.3 million according to Ministry of Education figures from February 2014) over the subsequent six years, in accordance with survey results. Six years was chosen as WHO recommends re-assessment surveys after 5–6 years [6]. Using WHO guidelines, we assumed low endemicity districts would be treated every three years (such that SAC are treated twice during primary schooling), moderate endemicity districts would be treated biennially and high endemicity districts would be treated annually, while districts with prevalence below 1% would not receive treatment.

Under the assumption that treating districts more frequently than required is not harmful but treating less frequently is, we define districts as receiving “adequate” treatment when they are assigned to either their correct or a higher treatment class. The annual cost per district adequately treated, c, incorporates the assumptions described and was calculated using Eq 1. We converted c to a percentage of the annual cost of blanket treatment per district (*c*_prop_), using Eq 2. Cost-efficiency is optimised when *c*_prop_ is minimised. Parameters are defined in Table 4.

**Table 4:** Definitions of parameters used in cost-efficiency equations.

Parameter	Verbal Description	Calculation
S	Cost of survey	
N	Number of school-aged children in country	
t	per capita cost of treatment and its delivery	
R	Mean (average across districts) number of rounds of treatment given over a 6 year period according to survey results	
T	Cost of nationwide treatment for 6 years based on survey results	N*t*R
P	Mean proportion of times districts were adequately treated in simulations	
D	Number of districts nationwide	
B	Annual cost of blanket treatment per district	(N*t)/D

$$c=\frac{1}{6}\frac{\left(S+T\right)}{P.D}$$ (1)

$$c_{prop}=100.\frac{c}{B}$$ (2)

The sensitivity of survey costs to variation in three inputs was assessed: 1) the number of survey teams, 2) survey staff salaries and 3) capital item lifespan. We also examined how variation in per capita treatment cost affected cost-efficiency, as per-capita treatment costs are likely to vary according to country-specific delivery costs and economies of scale [28,30]. Finally, to explore how conclusions might be affected by country-specific differences in geography and epidemiology, we examined cost-efficiency using simulation results for either Côte d’Ivoire or Liberia, combined with Malawi’s survey cost estimates and SAC population size.

## Results

### Spatial heterogeneity in schistosomiasis prevalence

**Fig 2:**
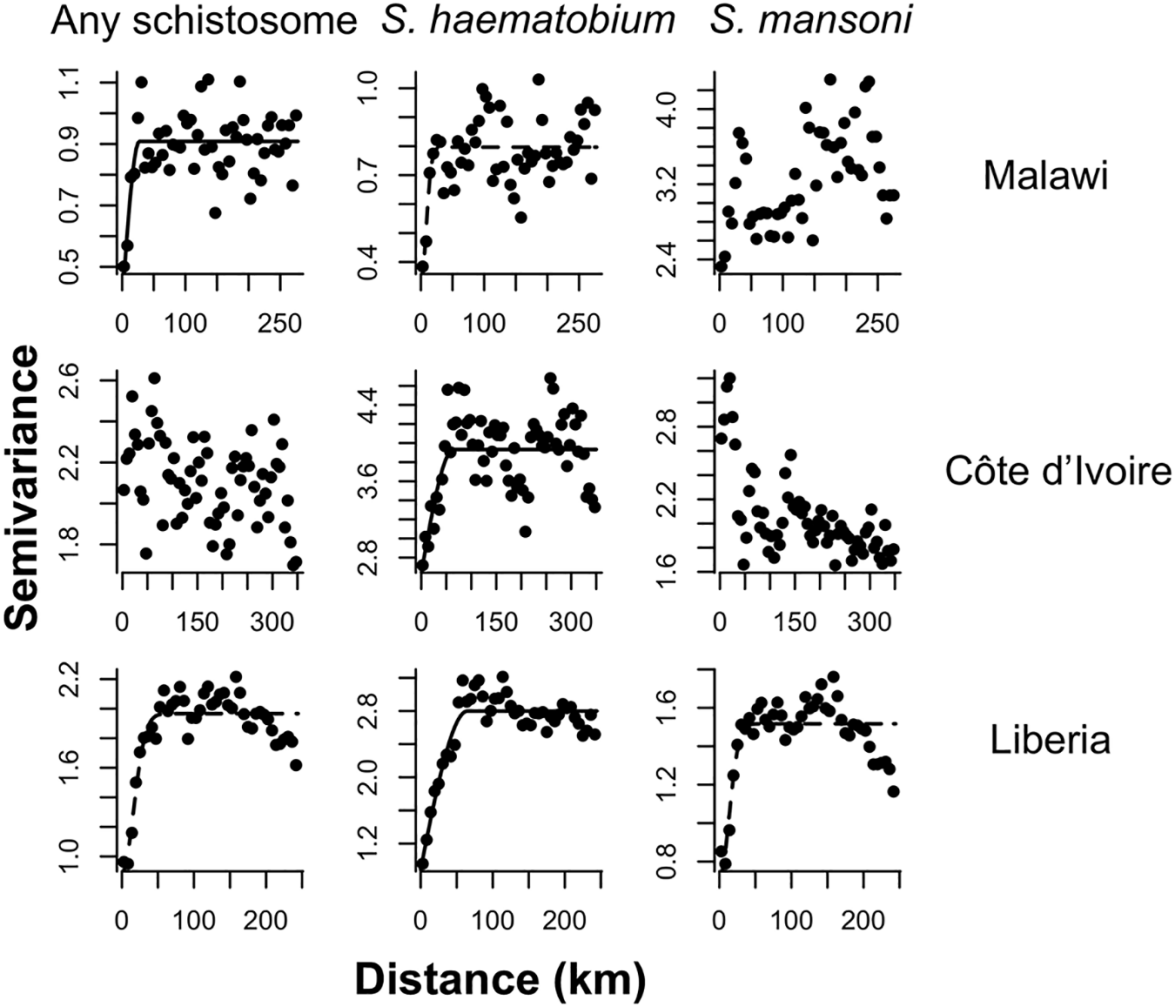
Omnidirectional semivariograms for schistosomiasis prevalence across primary schools in Malawi, Côte d’Ivoire and Liberia. Best fit lines of exponential (solid line) or Gaussian (dashed line) spatial models are shown and where no line is shown no significant spatial autocorrelation was detected. Distance in kilometres was calculated assuming 1 decimal degree is approximately 111km (as at the equator).

**Fig 3:**
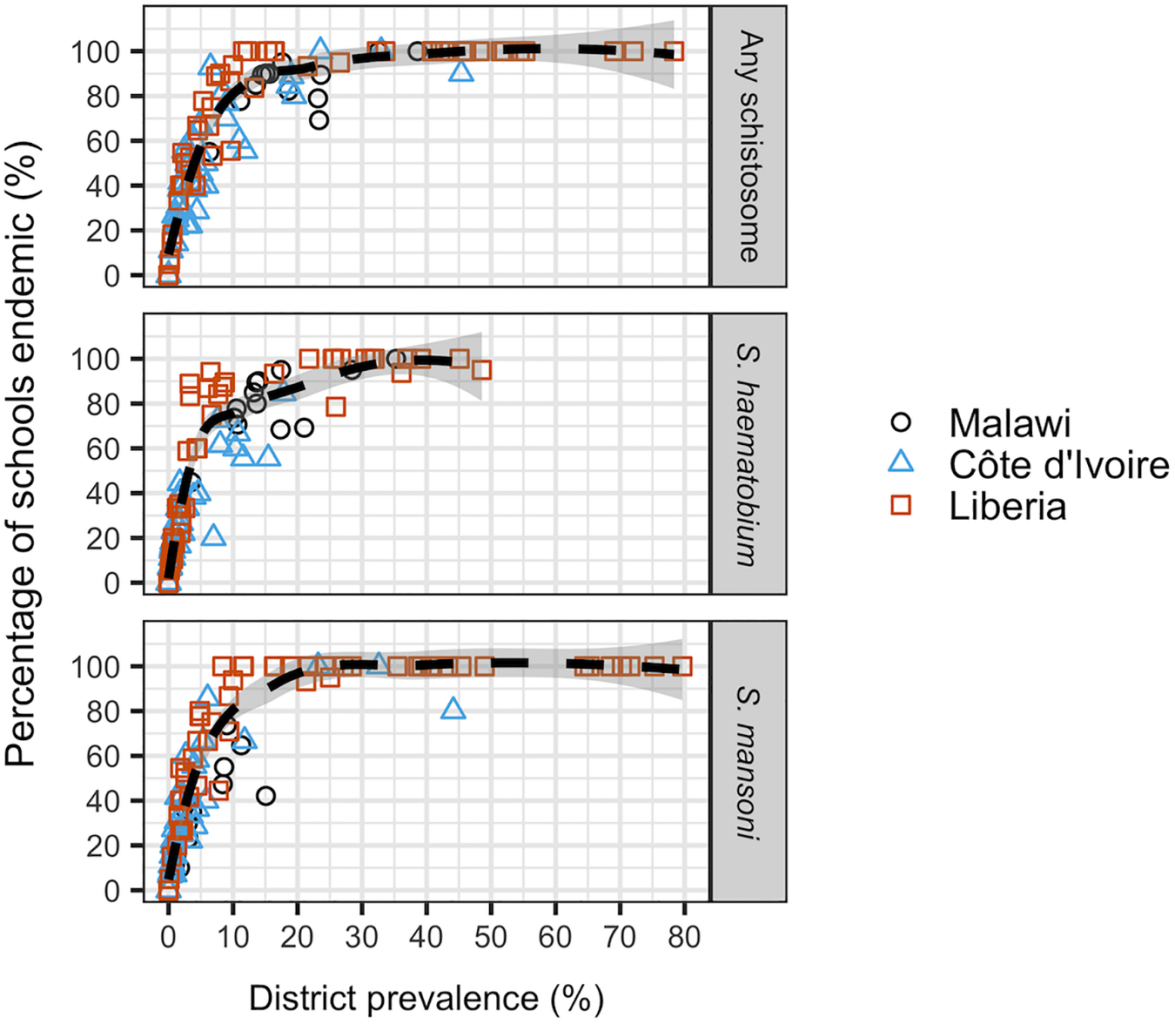
District-level relationship between prevalence and the percentage of schools endemic for schistosomiasis (any species), *S*. *haematobium* or *S*. *mansoni* in Côte d’Ivoire, Malawi and Liberia. Data were collected from mapping surveys using a standardised two-stage cluster survey design: random sampling of 15–20 schools per district, and 30 children at each school.

Countries varied in overall schistosomiasis prevalence, species composition and the spatial heterogeneity of prevalence (Table 2, Fig C in S3 Text). Spatial autocorrelation in prevalence was detected for *S*. *haematobium* in all three countries, for pooled schistosomiasis in Malawi and Liberia but not Côte d’Ivoire, and for *S*. *mansoni* only in Liberia (Fig 2). Range values indicated that no spatial correlation was present beyond 20–65km, with the exact distance within this range depending on the country and species considered. Values of the district-level intra-cluster correlation coefficient (ICC) ranged from 0 to 0.774 across the three country dataset (Table 5, Fig C in S3 Text), indicating that districts varied widely in the extent to which prevalence clustered by school. Districts of a given endemicity class (according to the point estimate of prevalence), contained schools with widely varying schistosomiasis prevalence, particularly in moderate and highly endemic districts (Fig D in S3 Text). A saturating relationship was seen between survey-estimated district prevalence and the proportion of schools positive for schistosomiasis; the proportion of endemic schools increased steeply up to ~10% prevalence, but once district prevalence exceeded 30% it was rare to find schools entirely free of infection (Fig 3).

**Table 5:** Mean and range of the district-level intra-cluster correlation coefficient (ICC) for schistosomiasis prevalence in Malawi, Côte d’Ivoire and Liberia, as estimated from empirical mapping survey datasets. N indicates the number of endemic mapping units (usually districts) included in the calculation.

Country	Pooled schistosomiasis		*S*. *haematobium*		*S*. *mansoni*	
	ICC mean (range)	N	ICC mean (range)	N	ICC mean (range)	N
**Malawi**	0.129 (0.015–0.382)	13	0.110 (0.016–0.372)	13	0.333 (0.013–0.751)	13
**Côte d’Ivoire**	0.176 (0–0.697)	41	0.150 (0–0.774)	36	0.192 (0–0.751)	40
**Liberia**	0.039 (0–0.263)	55	0.080 (0–0.680)	44	0.037 (0–0.322)	53

### Survey performance: Accuracy

**Fig 4:**
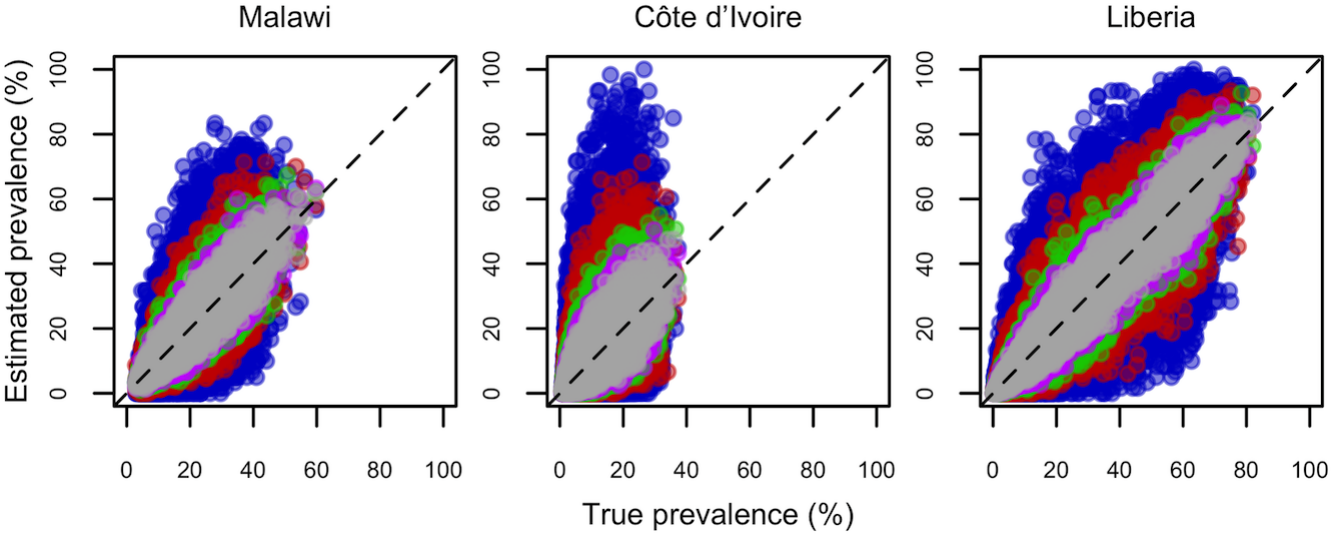
Plots illustrating the relationship between estimated and true schistosomiasis prevalence across 1000 simulated realisations for Malawi, Côte d’Ivoire and Liberia. All data relate to a sample size of 30 children per school, and colours represent the number of schools sampled per district (blue = 2, red = 5, green = 10, pink = 15, grey = 20). Each dot represents a realisation, and dashed lines indicate perfect correspondence between true and estimated prevalence. Points from surveys of different sizes are overlaid, from smallest (n = 2 schools, blue points at the back) to largest (n = 20 schools, grey points at the front).

**Fig 5:**
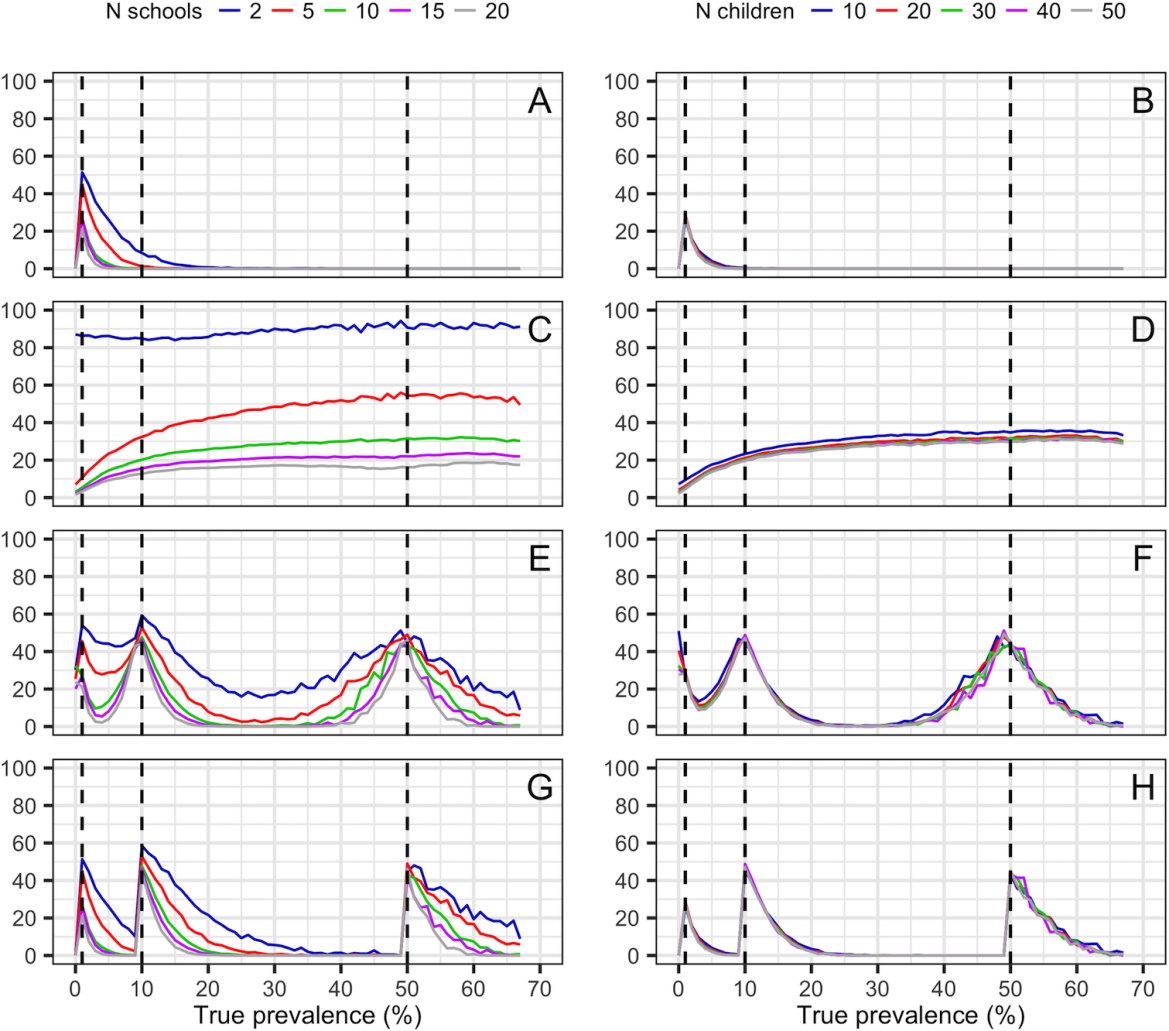
The effect of survey design on district-level schistosomiasis treatment decisions, using gold standard simulated data for Malawi, Côte d’Ivoire and Liberia. Left panels (A, C, E, G) show the effect of altering the number of schools sampled, while holding the number of children sampled constant at 30 per school, while right panels (B, D, F, H) show the effect of altering the number of children tested at each school, while holding the number of schools visited constant at 10 per district. Dashed lines indicate thresholds between low, moderate and high endemicity treatment classes according to WHO guidelines (Table 1). Lines indicate mean values for each survey design across the full three country gold standard dataset. A and B: the proportion of times a survey failed to detect endemic schistosomiasis (≥1% prevalence); C and D: the width of the 95% confidence interval around a district-level prevalence estimate; E and F: the proportion of times districts were wrongly classified into either a higher or lower treatment class; G and H: the proportion of times districts were classified into a treatment class below their true class.

District prevalence estimates converged on true prevalence as the number of schools surveyed increased (Fig 4), as expected. The probability of failing to detect endemic schistosomiasis at treatable levels (≥1% prevalence) declined as more schools were sampled per district (though with diminishing returns, particularly beyond 10 schools per district), while increasing the number of children tested per school led to very small improvements (Fig 5A and 5B, Fig E in S3 Text and Table D in S2 Text). Failure to detect treatable levels of schistosomiasis with smaller surveys was most acute in districts with low prevalence. For example, surveying 2–5 schools in low endemic districts led to detection failure 10–50% of the time (Fig 5A, Table D in S2 Text). Surveying more schools clearly improved the precision of prevalence estimates and the accuracy of treatment class assignment (Fig 5C, 5E and 5G, Fig E in S3 Text), again with diminishing returns above 10 schools per district. In contrast, increasing the number of children sampled per school led to negligible improvements in precision and assignment accuracy (Fig 5D, 5F and 5H, Fig E in S3 Text). For a given total sample size, sampling fewer children in more schools rather than many children in few schools minimised detection failure and maximised the accuracy of prevalence estimates and treatment class assignment (Fig F in S3 Text).

The use of more lenient district assignment rules (boosting rules) reduced the probability that districts were classified into a treatment class below their true class (Fig G in S3 Text and Table E in S2 Text). Use of boosting rules also allowed smaller surveys to be used while still achieving a specified maximum allowable probability of under-classification. For example, if one wanted the risk of district under-classification to not exceed 7%, this could be achieved by either surveying 20 schools and using point estimate district assignment, or surveying 10 schools and using a 2% boosting rule. District size had an influence on some measures of survey accuracy, with the probability of wrongly classifying and under-classifying districts being greater in large districts, for a given survey design (Fig H in S3 Text). However, district size did not appreciably alter the shape of the relationship between survey size and measures of accuracy (Fig H in S3 Text).

Although surveying more schools per district improved the accuracy of district-level classification, schools still showed great variation in prevalence within any given district. If the same WHO thresholds were applied at a school level, under most survey designs around 50% of schools would have been wrongly classified and 10–30% under-classified, with only very modest improvement as more schools were sampled per district (Fig I in S3 Text). This is due to the underlying large heterogeneity in prevalence among schools within a district (Fig D in S3 Text).

### Survey performance: Cost efficiency

**Fig 6:**
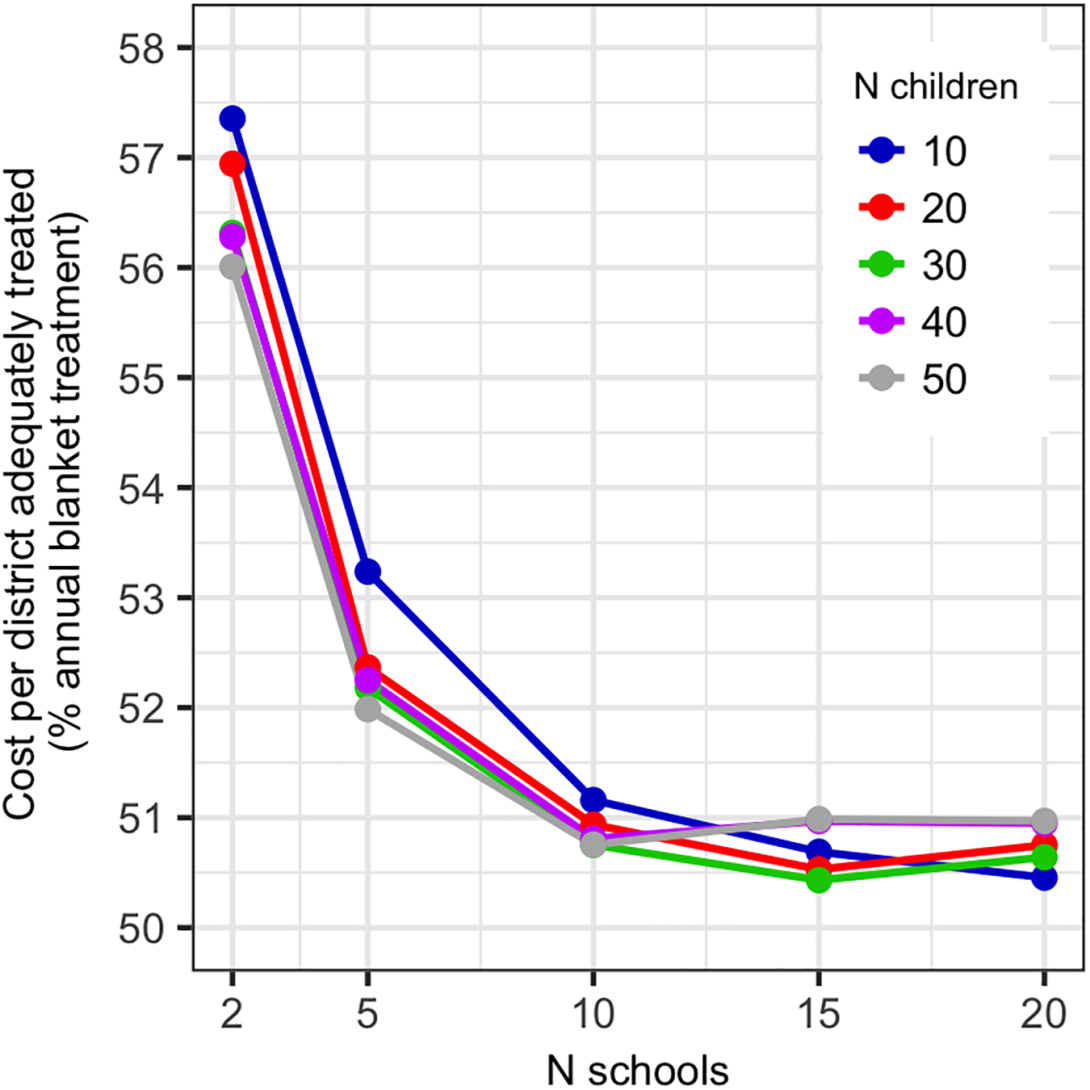
Effect of altering the number of schools surveyed per district and the number of children sampled per school in schistosomiasis surveys on the cost per district adequately treated (cost-efficiency is maximised when this is at a minimum).

**Fig 7:**
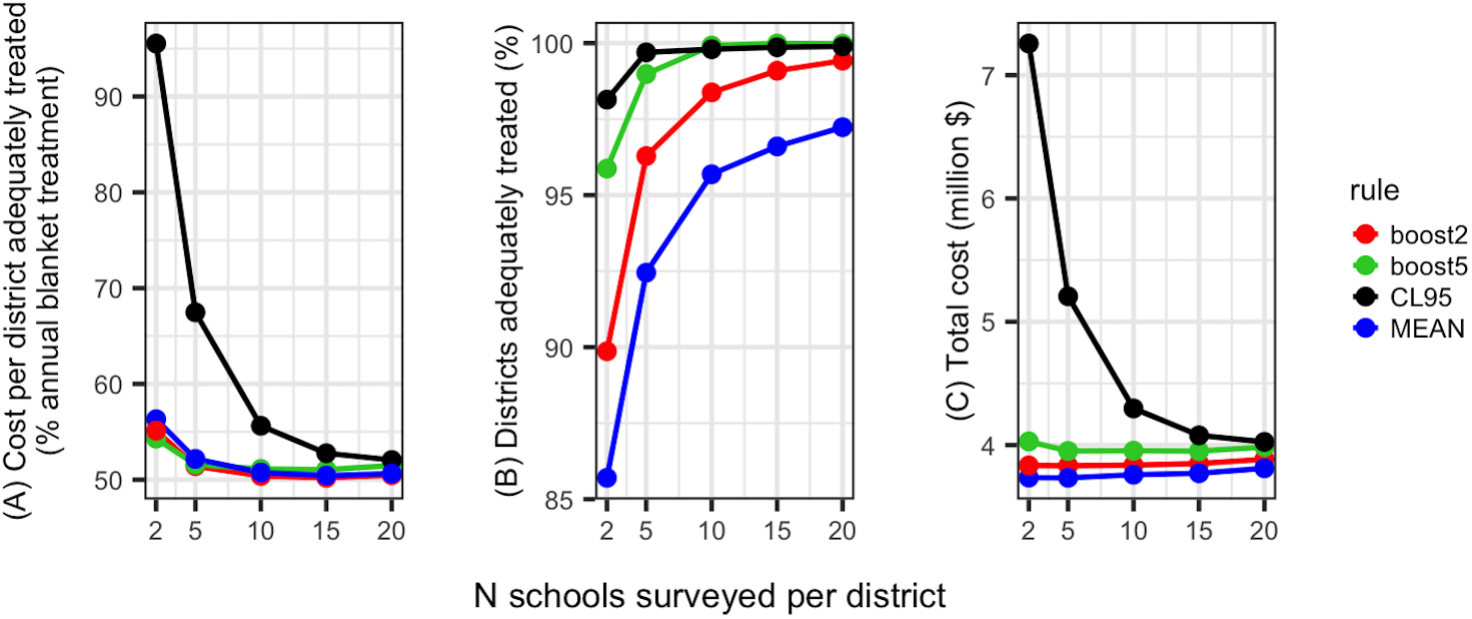
The effect of schistosomiasis survey size (number of schools surveyed per district) and the rule used for converting district-prevalence estimates into treatment class assignments on (A) the cost per district adequately treated (cost-efficiency is maximised when this is at a minimum), (B) the proportion of times a district was adequately treated and (C) combined survey and subsequent treatment costs. Line colour indicates the district classification rule used (blue: point prevalence estimate; red: 2% boost at thresholds, green: 5% boost at thresholds, black: upper 95% confidence limit). All plots relate to surveys where 30 children were sampled per school.

Based on a range of 2–20 schools surveyed per district and 10–50 children sampled per school, the estimated cost of a nationwide schistosomiasis survey in Malawi varied between $22,482 and $135,033 (Table F in S2 Text). In sensitivity analyses, survey costs were most sensitive to variation in staff salaries (per diems), while the number of survey teams and capital item lifespan had minimal effects (Fig J in S3 Text). The number of schools surveyed strongly influenced both the absolute cost of surveys and cost-efficiency, while varying the number of children screened per school had smaller cost implications, particularly in terms of cost efficiency (Fig 6, Fig K in S3 Text). Under parameters expected for Malawi (per capita treatment cost $0.30, 4.3 million SAC nationwide), surveying 15 schools per district was the most cost-efficient survey size, with assignment using the point prevalence estimate or boosting rules performing similarly (Fig 7A). Use of 95% confidence intervals to assign treatment classes was cost inefficient, particularly with small surveys where confidence intervals were wide (Fig 7A). While the use of boosting rules was not always the most cost-efficient strategy, they notably improved the proportion of districts adequately treated (Fig 7B) for some additional cost (Fig 7C).

**Fig 8:**
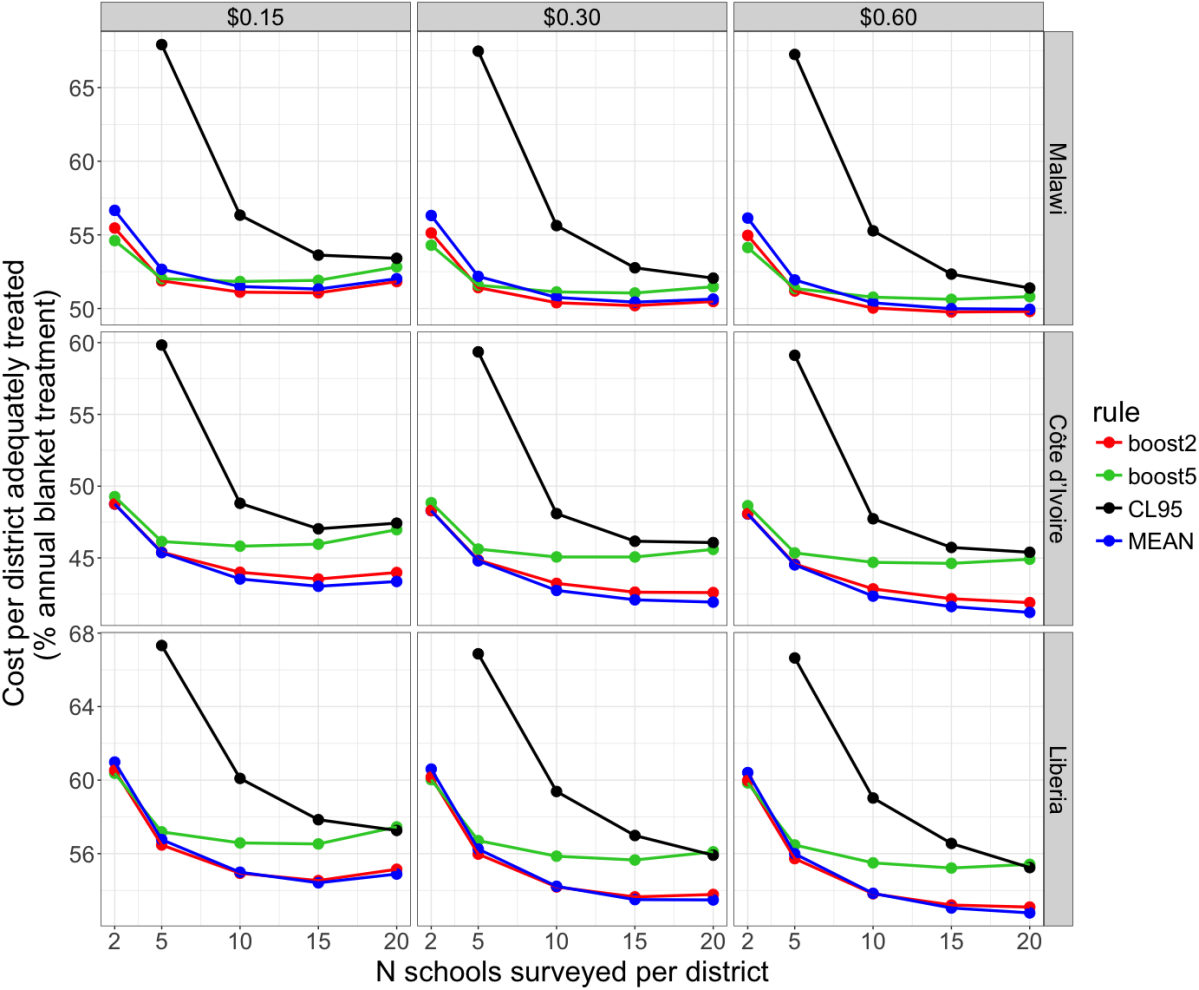
Effect of variation in per capita PZQ treatment costs (0.15 to 0.60 USD) and the country source of simulation results on estimates of mapping survey cost-efficiency. Cost-efficiency is expressed as the cost per district adequately treated, as a percentage of the annual blanket treatment cost for a district. Line colour indicates the district classification rule used (blue: point prevalence estimate; red: 2% boost at thresholds, green: 5% boost at thresholds, black: upper 95% confidence limit). All plots relate to surveys where 30 children were sampled per school. Data points for surveys using 2 schools per district and assignment using the upper 95% confidence limit (which all produced very high values, >90% on the y-axis) are not shown, to improve clarity in the lower range of the y-axis.

These cost-efficiency results for Malawi were robust to variation in staff salaries (Fig L in S3 Text). Furthermore, when we halved or double the assumed *per capita* treatment cost, and assessed how cost-efficiency might be altered when simulation results from either Côte d’Ivoire or Liberia were used instead of those from Malawi, results were remarkably consistent: surveys of 15–20 schools per district maximised cost-efficiency in all cases (Fig 8). As treatment costs increased, the cost-efficiency of surveying more schools per district increased, and at $0.60 per treatment a survey of 20 schools per district was often more cost efficient than 15 (Fig 8). In these sensitivity analyses, treatment class assignment using either the point prevalence estimate or the two percentage point boosting rule was most cost efficient (depending on the country considered), with worse cost-efficiency for the larger five percentage point boosting rule, and worse still when using 95% confidence intervals (Fig 8).

## Discussion

Prevalence surveys form a key component of schistosomiasis control programmes, allowing programmes to guide delivery of praziquantel to those areas most in need of treatment. Using in silico sampling simulations based on data from three African countries, we evaluated the performance of cluster survey designs differing in the sample size at each of two stages (schools and children within schools) and a series of rules to translate resulting prevalence estimates into treatment class assignments. Overall, our results provide an evidence base for schistosomiasis programmes wishing to map prevalence, illustrating how different two-stage cluster survey designs impact the accuracy and cost-efficiency of treatment decisions.

### Spatial heterogeneity in schistosomiasis prevalence

Schistosomiasis is often described as a focal disease, with prevalence varying widely even from one village to the next. Our results from Malawi, Côte d’Ivoire and Liberia illustrate this well. Prevalence varied very widely across schools in moderate or high endemicity districts (Fig D S3 Text), and spatial autocorrelation in prevalence was detected across short distances (20–65km), similar to previous findings from East and West Africa [15,31]. Moreover, spatial autocorrelation was not detected in all cases, indicating no spatial clustering or clustering at scales too small to detect with our data. District-level intracluster correlation coefficients (ICC) were also highly variable, ranging from 0 to 0.775 with a mean of 0.116. These findings indicate greater within-district spatial heterogeneity in schistosomiasis prevalence than reported for STH [16].

### Comparative survey performance

Our simulations clearly demonstrated that increasing the number of schools surveyed provided much greater gains in the accuracy of prevalence estimates and treatment class assignments than increasing the number of children tested per school. Surveying too few schools per district risked failing to detect treatable levels of schistosomiasis, particularly in low endemic districts. For instance, simulated surveys of five schools per district led to low endemic districts being classified as non-endemic between 2% and 23% of the time. Since district prevalence will usually not be re-assessed for 5–6 years [6], it is important that enough schools are surveyed initially such that districts are not erroneously classified as non-endemic, missing treatment for several years.

Using cost data from Malawi, cost-efficiency improved notably as surveys increased in size from 2 to 15 schools per district, with only a very small decline in cost-efficiency at 20 schools per district. Conversely, the number of children sampled per school did not greatly affect cost-efficiency. Decisions about sample size per school could, therefore, be made in light of practical considerations, such as to maximise the number of schools visited per day, provide reasonably accurate prevalence estimates to feed back to communities, or acquire data for operational research needs. Sensitivity analyses showed that as treatment costs increased, larger surveys of 20 schools per district became most cost-efficient; essentially, the higher the cost of nationwide treatment, the greater the benefit of larger surveys to enable accurate geographic targeting of drug delivery. This may apply even more so in suspected high endemicity areas where treatment of at-risk adults as well as SAC is to be performed. In all sensitivity analyses, surveys involving 10 or fewer schools per district showed inferior cost-efficiency compared to those involving 15–20 schools. Thus, although surveys of five schools per district have been suggested to be cost efficient for soil-transmitted helminths [16], schistosomiasis surveys need to be larger [10]. Two-stage cluster surveys aiming to provide treatment guidelines for both types of infection should therefore optimise sample size for schistosomiasis, and STH prevalence estimates of sufficient accuracy will follow.

The latest draft of WHO guidelines on schistosomiasis mapping [32] suggest that districts can be mapped with as few as five schools per district, where the distribution of infection is thought to be homogeneous. Our results suggest that this may not be optimal, as this sample size led to some districts with treatable levels of infection going undetected, and poorer cost-efficiency than surveys involving 15 or 20 schools per district. Thus, what might be perceived as quite large surveys (15–20 schools per district) are shown here to pay off for schistosomiasis mapping in terms of cost-efficiency. The draft guidelines also suggest surveying 50 children per school. Our results indicate that a sample size of 20–30 children per school produced very similar accuracy and cost-efficiency results to those involving 50 per school (Fig 4, Fig F in S3 Text), such that this recommended within-school sample size could be reduced, particularly when paired with surveying a larger number of schools (e.g. 15–20) per district.

We found that using alternative rules to convert prevalence estimates into treatment class assignments could be beneficial in some situations. The use of boosting rules, where districts were boosted into the next highest treatment class when close to a threshold, in combination with small surveys (2–5 schools per district) was associated with a reduced risk of under-treatment, and comparable or improved cost-efficiency compared to assignment using point prevalence estimates. However, with larger surveys (10–20 schools) this pattern reversed and while boosting rules reduced under-treatment they also reduced cost-efficiency. Therefore, when small surveys (<10 schools per district) are unavoidable due to logistical, budgetary or time constraints, boosting rules might prove useful to avoid under-treatment. Use of 95% confidence intervals in assignment of districts to treatment classes was never cost-efficient due to a high degree of over-treatment. This was particularly true when used in combination with small survey sizes, suggesting this practice should be avoided.

By using data from three different African countries, our simulations allowed us to identify a ball-park sample size and design for optimising district-level schistosomiasis surveys across similar settings. However, several limitations to this study warrant discussion. First, we only compared simple two-stage cluster surveys, frequently adopted where there is limited reliable information about schistosomiasis distribution that could be used to design more complex, potentially more powerful surveys, or when a survey design that is easy to implement and analyse is desired. If information about the likely distribution of infection is available and is of sufficient quality and geographic scope, other survey designs could prove superior. For example, where schistosomiasis prevalence is strongly associated with known geographic risk factors (such as lake proximity for *S*. *mansoni* in Uganda and Burundi [33]), geographic survey stratification can improve the accuracy of district-level prevalence estimates, while guarding against random samples that happen not to capture schools in high risk areas. Re-assessment surveys may often be able to incorporate such stratification, using prevalence information from prior surveys to guide design. Where geographic risk factors drive strong within-district variation in prevalence, survey and treatment strategies at a sub-district level are also likely to be fruitful, where feasible. If geographic coordinates of potential sampling locations are available, explicitly spatial survey designs may also be an option [15,34] and geospatial modeling can be used to convert survey data into PC implementation maps [34–36]. However, both stratified designs and geospatial approaches require increased expertise for appropriate survey implementation and data analysis. Studies examining how best to optimise other surveys designs, for example those stratifying for water body proximity for *S*. *mansoni*, would be valuable. Second, we have not thoroughly examined how differences in factors such as district size, population density and urbanization, all of which can vary widely across countries, might affect optimal survey design. We show that the accuracy of district assignment to treatment classes was lower for larger districts (Fig H in S3 Text). However, since survey costs can also be expected to scale with the size of districts, it is unclear exactly how district size might alter survey cost-efficiency. With cost data from only one country we were unable to explore this. Future studies that do so, using matched survey and cost data from a range of countries with pronounced variation in factors like district size, would be valuable. Finally, while our results concern the use of urine filtration and Kato-Katz for diagnosing schistosome infection, use of the urine-based circulating cathodic antigen (CCA) test for *S*. *mansoni* surveys is increasing [37,38]. It is possible that use of this alternative diagnostic could alter optimal survey size, for example if increased test sensitivity leads to lower observed spatial heterogeneity in infection. Thus, our findings may not be directly applicable to the use of different diagnostic tests, and further work is needed to understand how the CCA test alters survey results and optimal design.

Although our aim here was to assess survey performance for generating district-level treatment decisions, our data highlight the broader issue that schools within a district often vary widely in prevalence, particularly in high prevalence areas (Fig D in S3 Text). If WHO treatment recommendations were applied at the school level, our simulations showed that using district level prevalence estimates derived from two-stage cluster surveys would lead to 10–30% schools being under-treated. Increasing survey size made very limited improvements here, and achieving this would require a fundamental shift in approach, for example towards either surveys at finer spatial scale or school-level surveillance. Currently, the cost and complexity of schistosomiasis diagnostic tests means surveillance is often impractical, particularly for *S*. *mansoni* where a test that could be easily administrated by teachers is not currently available. In areas where only *S*. *haematobium* is endemic, however, surveillance strategies may be fruitful and could for example utilise blood-in-urine questionnaires or urine dipsticks [39] delivered to schools through widespread vaccination or STH (Albendazole) control programmes, such that they could self-perform and report an LQAS assessment for schistosomiasis. Alternatively, geospatial modeling could be used to predict endemicity class at non-surveyed schools using data from surveyed schools [15]. Such school-level approaches could ameliorate the problem highlighted here that district-level averages mask wide variation in infection risk among sites, though use of such approaches would require new accompanying guidelines for converting site-specific prevalence levels into treatment practice.

## Conclusion

Based on our findings, we suggest that among simple two-stage cluster survey designs, sampling 15–20 schools per district and 20–30 children sampled per school is suitable for estimating district-level schistosomiasis prevalence and placing districts into treatment classes. This design fulfilled three key requirements: (1) it minimized the risk of failing to detect treatable infection levels in a district (2) it generated reasonably accurate prevalence estimates and treatment decisions and (3) it was cost-efficient, minimizing the cost per district adequately treated. Surveys towards the upper end of this range are likely to be more cost-efficient where treatment costs are high. The data presented here provides an initial evidence base for sample size in schistosomasis surveys, which can be built upon by future work assessing optimal survey design in the context of disease control. Our approach can also be adapted to inform on optimal survey design for other NTDs where two-stage cluster surveys are appropriate.

## Supporting information

S1 Text Additional methods (incl. sample size calculations) for baseline mapping surveys. (DOCX)

S2 Text Supplementary tables. (DOCX)

S3 Text Supplementary figures. (DOCX)

S1 File Empirical survey data used in analysis. (DOCX)

S1 Appendix Strobe check list. (DOC)
