# Supplementary material for: Optimising cluster survey design for planning schistosomiasis preventive chemotherapy
Source: PLoS Negl Trop Dis. 2017 May 26;11(5):e0005599. doi: 10.1371/journal.pntd.0005599 (PMC5464666; doi:10.1371/journal.pntd.0005599)
Supplement: S1 Text — (DOCX) [file pntd.0005599.s001.docx]

**Schistosomiasis survey sample size calculations**

Sample size calculations were performed by the Biostatistician at the Schistosomiasis Control Initiative. Survey data from Uganda (*S. mansoni*) and Burkina Faso (*S. haematobium*) were first used to derive estimates of the intra-cluster correlation coefficient, ICC. The ICC was estimated at 0.33 for *S. mansoni* prevalence across a substantial area of Uganda and at 0.35 for *S. haematobium* prevalence across Burkina Faso. Since ICOSA surveys would estimate prevalence at a district level, and districts are likely to be less ecologically heterogeneous than these large geographic areas, we used an estimated ICC value of 0.2. Sample size calculations were performed to find a two-stage cluster survey design that would be capable of estimating 50% prevalence with a 10-percentage point margin of error on the 95% confidence interval, using calculations from Lohr (2009). This led to a recommended sample size of 20 schools per district, and 30 children sampled per school.

**References**

Lohr, S. 2009 *Sampling: design and analysis*. Cengage Learning.
