# Supplementary material for: Optimising cluster survey design for planning schistosomiasis preventive chemotherapy
Source: PLoS Negl Trop Dis. 2017 May 26;11(5):e0005599. doi: 10.1371/journal.pntd.0005599 (PMC5464666; doi:10.1371/journal.pntd.0005599)
Supplement: S2 Text — (DOCX) [file pntd.0005599.s002.docx]

**Supplementary tables**

**Table A:** Summary of country data used in generation of gold standard datasets. Only districts in which 20 or more school locations were assigned were included in simulations, such that all sampling strategies could be compared across all countries.

| **Country** | **N primary schools** | **Average SAC per school** | **N districts simulated**  **(N districts in country)** | **Data sources** |
| --- | --- | --- | --- | --- |
| Malawi | 5,239 | 400 | 26 (26) | ^1,2^ Fully geo-referenced primary school database ^3^ DIVA-GIS website |
| Côte d’Ivoire | 11,429 | 250 | 78 (82) | ^1^ Primary school list obtained from the Ministry of Health; ^2^ Enrolment figures collected in 8 districts during ICOSA mapping; ^3^ Heath district shapefile obtained from Ministry of Health |
| Liberia | 2,785 | 150 | 39 (66) | ^1, 2^ 2013 EMIS National Statistical Booklet; ^3^ DIVA-GIS website |

^1^ Source for number of primary schools nationwide

^2^ Source for average SAC per school

^3^ Source for district number and geographic boundaries

**Table B:** Assumed useful lifespan of capital equipment used in calculations of mapping survey costs.

| **Capital equipment* (Fixed)** | **Useful lifespan (years)** |
| --- | --- |
| Basins | 5 |
| Brushes | 2 |
| Buckets | 5 |
| Droppers | 2 |
| Forceps | 5 |
| GPS | 5 |
| Hole punch | 5 |
| Microscope | 5 |
| Sieving mesh | 3 |
| Slide boxes | 5 |
| Tally counters | 5 |
| **Capital equipment* (Variable)** |  |
| Filter Holders | 4 |
| Stool pots | 5 |
| Syringes | 5 |
| Kato-Katz kits (template and plastic spatula) | 4 |
| Urine Pots | 5 |

**Table C:** Estimated number of days required to survey a district under different mapping survey designs, based on field experience in Malawi. Survey duration estimates included a day for traveling to/from the field and account for the fact that school visits cannot occur on weekends.

|  |  | **Number of schools** | | | | |
| --- | --- | --- | --- | --- | --- | --- |
|  |  | **2** | **5** | **10** | **15** | **20** |
| **Number of children tested per school** | **10** | 2.1 | 3.1 | 4.1 | 5.1 | 6.1 |
|  | **20** | 2.1 | 3.1 | 5.1 | 6.1 | 10.1 |
|  | **30** | 2.1 | 3.1 | 5.1 | 6.1 | 10.1 |
|  | **40** | 2.1 | 4.1 | 6.1 | 11.1 | 13.1 |
|  | **50** | 2.1 | 4.1 | 6.1 | 11.1 | 13.1 |

# Table D: The proportion (%) of simulated surveys failing to detect treatable levels (≥1%) of schistosomiasis in simulated datasets, according to district-level prevalence classes (low: 1-10%; moderate: 10-50%, high: 50% and above).

|  |  | **Surveys failing to detect ≥1% schistosomiasis prevalence (%)** | | |
| --- | --- | --- | --- | --- |
| **N schools** | **N children** | ***Low*** | ***Moderate*** | ***High*** |
| 2 | 10 | 52.88 | 11.91 | 0.02 |
| 2 | 20 | 36.14 | 4.78 | 0.00 |
| 2 | 30 | 27.53 | 2.43 | 0.00 |
| 2 | 40 | 21.61 | 1.30 | 0.00 |
| 2 | 50 | 17.16 | 0.83 | 0.00 |
| 5 | 10 | 22.58 | 1.15 | 0.00 |
| 5 | 20 | 10.30 | 0.18 | 0.00 |
| 5 | 30 | 5.81 | 0.05 | 0.00 |
| 5 | 40 | 3.33 | 0.02 | 0.00 |
| 5 | 50 | 2.07 | 0.00 | 0.00 |
| 10 | 10 | 6.48 | 0.06 | 0.00 |
| 10 | 20 | 1.77 | 0.00 | 0.00 |
| 10 | 30 | 0.65 | 0.00 | 0.00 |
| 10 | 40 | 0.30 | 0.00 | 0.00 |
| 10 | 50 | 0.12 | 0.00 | 0.00 |
| 15 | 10 | 2.12 | 0.00 | 0.00 |
| 15 | 20 | 0.38 | 0.00 | 0.00 |
| 15 | 30 | 0.11 | 0.00 | 0.00 |
| 15 | 40 | 0.03 | 0.00 | 0.00 |
| 15 | 50 | 0.01 | 0.00 | 0.00 |
| 20 | 10 | 0.82 | 0.00 | 0.00 |
| 20 | 20 | 0.11 | 0.00 | 0.00 |
| 20 | 30 | 0.02 | 0.00 | 0.00 |
| 20 | 40 | 0.00 | 0.00 | 0.00 |
| 20 | 50 | 0.00 | 0.00 | 0.00 |

**Table E:** Proportion (%) of times that simulated surveys placed districts in a treatment class below their true class, according to survey design and the rule used in district assignment.

| **N schools** | **N children** | **Point estimate** | **2% boost** | **5% boost** | **95% confidence limit** |
| --- | --- | --- | --- | --- | --- |
| 2 | 10 | 40.19 | 40.19 | 33.35 | 32.62 |
| 2 | 20 | 32.33 | 32.33 | 24.94 | 20.95 |
| 2 | 30 | 28.07 | 25.57 | 20.48 | 15.99 |
| 2 | 40 | 25.15 | 23.35 | 17.51 | 12.64 |
| 2 | 50 | 22.99 | 19.99 | 15.19 | 10.23 |
| 5 | 10 | 20.77 | 18.07 | 15.65 | 12.20 |
| 5 | 20 | 14.56 | 11.65 | 8.05 | 6.43 |
| 5 | 30 | 17.02 | 14.04 | 10.77 | 4.15 |
| 5 | 40 | 14.35 | 11.34 | 7.69 | 2.97 |
| 5 | 50 | 15.85 | 12.81 | 9.40 | 2.45 |
| 10 | 10 | 9.85 | 7.17 | 4.60 | 3.88 |
| 10 | 20 | 9.31 | 6.60 | 4.04 | 1.57 |
| 10 | 30 | 8.98 | 6.22 | 3.64 | 1.09 |
| 10 | 40 | 8.83 | 6.06 | 3.54 | 0.96 |
| 10 | 50 | 8.61 | 5.83 | 3.44 | 0.90 |
| 15 | 10 | 8.86 | 6.25 | 4.27 | 1.45 |
| 15 | 20 | 6.89 | 4.20 | 2.27 | 0.65 |
| 15 | 30 | 7.10 | 4.39 | 2.54 | 0.58 |
| 15 | 40 | 6.50 | 3.85 | 2.00 | 0.59 |
| 15 | 50 | 6.72 | 4.00 | 2.20 | 0.59 |
| 20 | 10 | 6.07 | 3.50 | 1.90 | 0.68 |
| 20 | 20 | 5.46 | 2.94 | 1.45 | 0.38 |
| 20 | 30 | 5.22 | 2.77 | 1.35 | 0.39 |
| 20 | 40 | 5.07 | 2.57 | 1.25 | 0.43 |
| 20 | 50 | 5.06 | 2.50 | 1.17 | 0.44 |

**Table F:** Estimated cost (in $ USD) of different surveys in Malawi covering all 26 districts, according to the number of schools visited per district and the number of children sampled per school.

|  |  | **Number of schools** | | | | |
| --- | --- | --- | --- | --- | --- | --- |
|  |  | **2** | **5** | **10** | **15** | **20** |
| **Number of children tested per school** | **10** | 22,482 | 32,916 | 43,468 | 53,896 | 64,571 |
|  | **20** | 22,800 | 33,626 | 53,372 | 64,452 | 101,409 |
|  | **30** | 23,118 | 34,335 | 54,734 | 66,465 | 104,074 |
|  | **40** | 23,437 | 43,588 | 64,638 | 111,194 | 132,368 |
|  | **50** | 23,755 | 44,297 | 65,999 | 113,207 | 135,033 |
