# Supplementary material for: Optimising cluster survey design for planning schistosomiasis preventive chemotherapy
Source: PLoS Negl Trop Dis. 2017 May 26;11(5):e0005599. doi: 10.1371/journal.pntd.0005599 (PMC5464666; doi:10.1371/journal.pntd.0005599)

**Supplementary figures**

**Figure A**: Comparison of true primary school locations (A and B) with those estimated using population-based approximations (C and D) in Malawi and Kenya respectively.


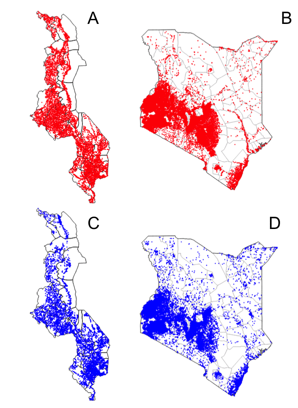


**Figure B**: Correlation between the number of real primary schools and the number of primary school locations simulated using a population-based approximation across 0.3 decimal degree grid squares in (A) Malawi and (B) Kenya.


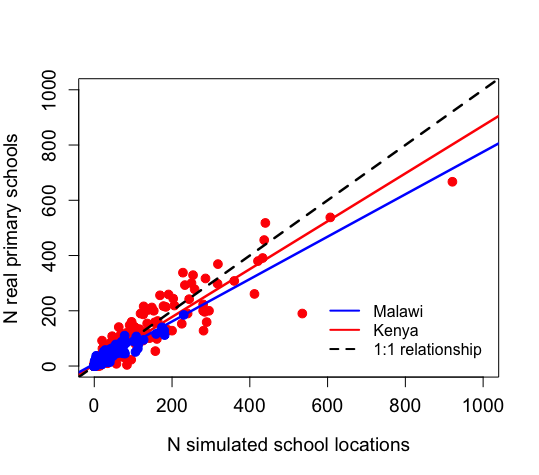


**Figure C:** Histograms illustrating variation in A) schistosomiasis prevalence across schools and B) district level intra-class correlation coefficients (ICC) for schistosomiasis prevalence, in empirical mapping datasets from Cote d’Ivoire, Malawi and Liberia.

**
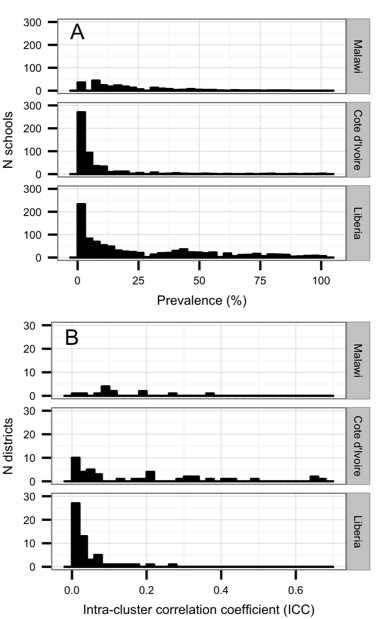
**

**Figure D:** Violin plots illustrating the range of school prevalence values for schistosomiasis (any species) in each district within baseline mapping data. Black diamonds indicate the district prevalence estimate, and horizontal dashed lines mark the WHO endemicity/treatment thresholds for schistosomiasis PCT (1-10% low endemicity, 10-50% moderate endemicity, 50% and above high endemicity). Wide variation among schools within a district of a given endemicity class is evident.

**
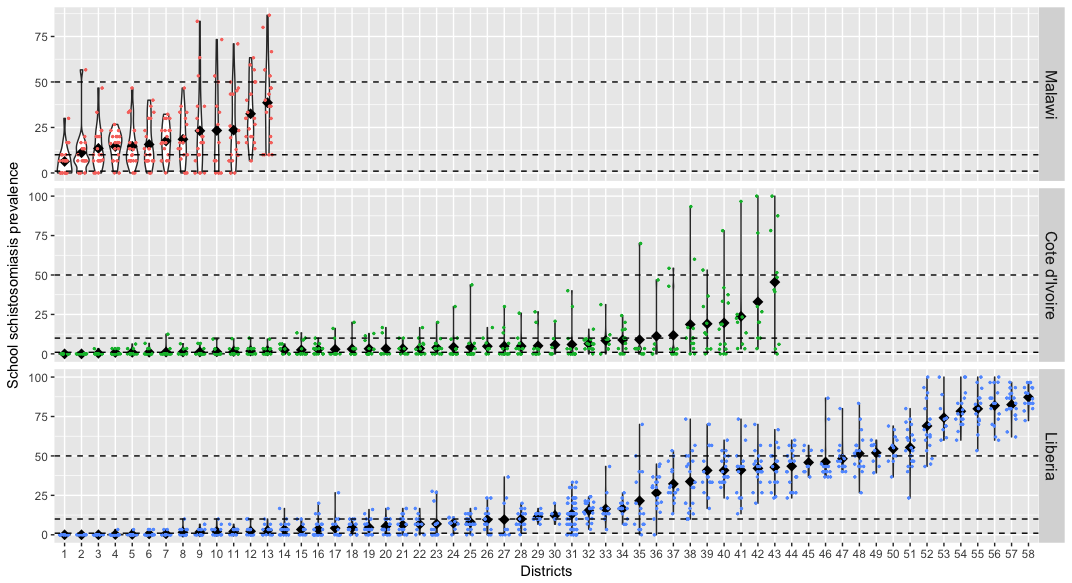
**

**Figure E:** The effect of survey design on the accuracy of district-level schistosomiasis treatment decisions, using a gold standard simulated dataset of 143 districts from Malawi, Côte d’Ivoire and Liberia. A) the proportion of times a survey failed to detect endemic schistosomiasis, B) the proportion of times districts were wrongly classified into either a higher or lower treatment class, and C) the proportion of times districts were classified into a treatment class below their true class.


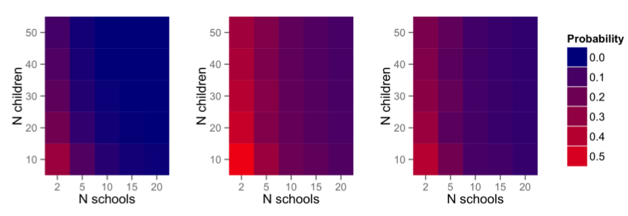


**Figure F**: The effect of mapping survey design on the accuracy of schistosomiasis treatment decisions for a given total sample size per district from simulations. (A) the proportion of times surveys failed to detect endemic schistosomiasis in districts (B) precision of district-level prevalence estimates, reflected by the mean width of exact 95% confidence intervals (C) the proportion of times districts were classified into the wrong endemicity category (D) the proportion of times districts were classified into an endemicity category below their true class.


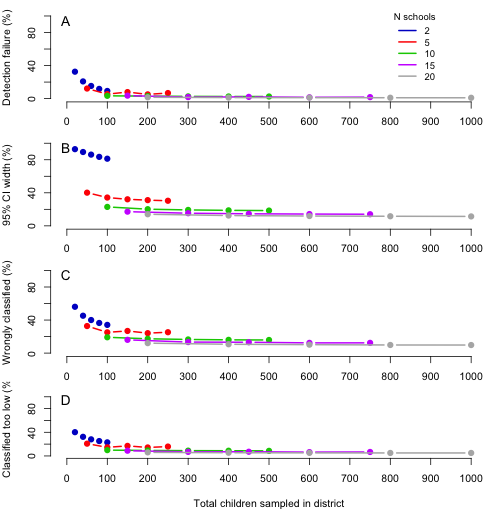


**Figure G**: The effect of alternative assignment rules on the probability of classifying districts into schistosomiasis endemicity classes below than their true class. In all graphs, the number of children sampled per school was kept constant at 30. The following four rules were considered for using prevalence estimates to classify districts (A) classify using the unadjusted point prevalence estimate (B) boosting districts where the point estimate is within 2 percentage points of the moderate or high endemicity class thresholds (C) boosting districts where the point estimate is within 5 percentage points of the moderate or high endemicity class thresholds (D) classify into a higher endemicity class if the upper 95% confidence limit overlaps that threshold.


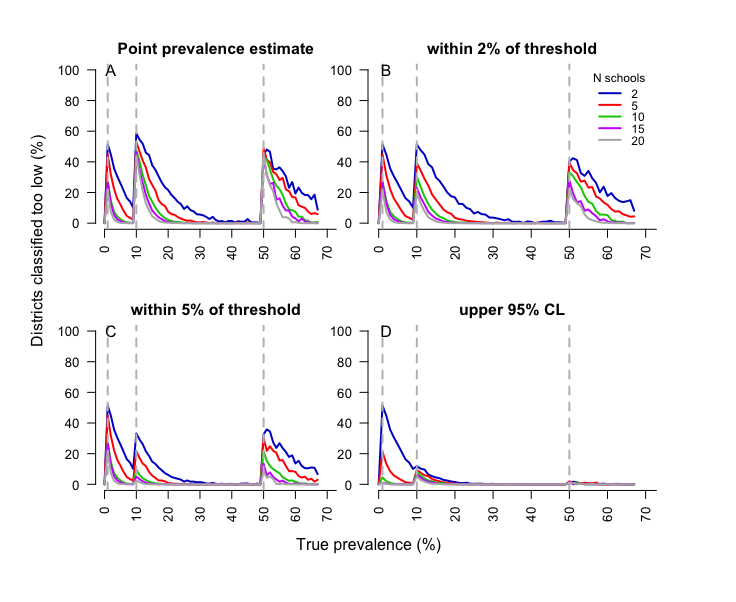


**Figure H:** Relationship between four measures of survey accuracy from simulations, and district size. Districts are grouped into size tertiles, small (<1438km^2^), medium (1439-4394km^2^) and large (>4395km^2^). Data are plotted for surveys involving 30 children sampled per school.

**
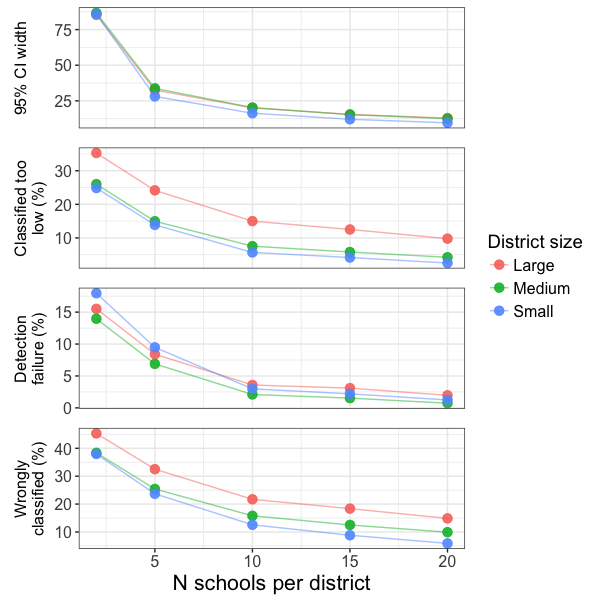
**

**Figure I**: Simulation results illustrating the proportion of times schools would have been (A) wrongly classified or (B) classified into an endemicity class below their true class, according to school-level prevalence under varying survey design.

**
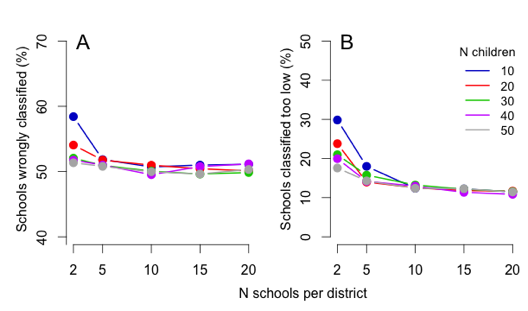
**

**Figure J**: Sensitivity (percentage change) of total survey cost to changes in three key cost drivers: Capital item lifespan (±20%), survey staff salaries (±20%) and the number of teams simultaneously performing the survey (1 to 5).


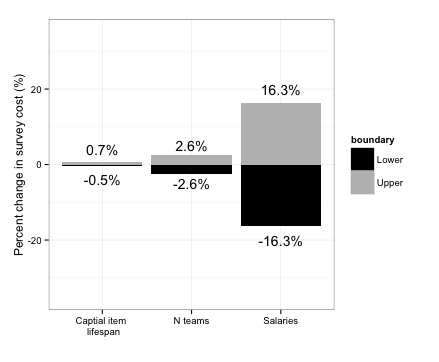


**Figure K:** Effect of the number of schools surveyed per district, and the number of children sampled at each school, on the estimated total cost of a mapping survey in Malawi.

**
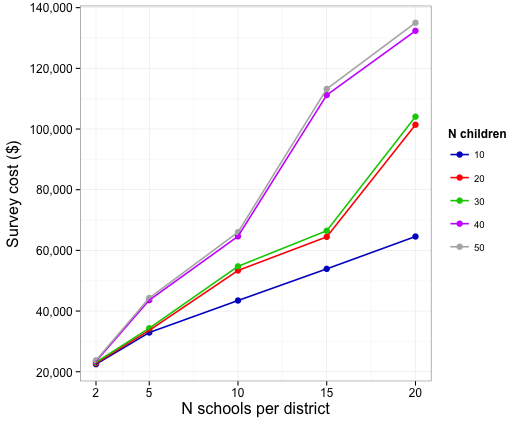
**

**Figure L:** Variation in survey team salary had almost no effect on the relative cost efficiency of alternate survey designs in Malawi. Line colour represents district classification rules (blue: point prevalence estimate; red: point prevalence with 2 percentage point boost at thresholds; green: point prevalence with 5 percentage point boost at thresholds; black: upper 95% confidence limit).


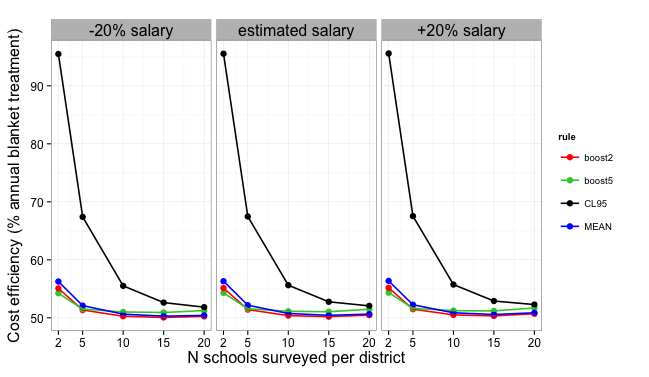

Supplement: S3 Text — (DOCX) [file pntd.0005599.s003.docx]
