# Supplementary material for: Optimising cluster survey design for planning schistosomiasis preventive chemotherapy
Source: PLoS Negl Trop Dis. 2017 May 26;11(5):e0005599. doi: 10.1371/journal.pntd.0005599 (PMC5464666; doi:10.1371/journal.pntd.0005599)
Supplement: S1 File — (DOCX) [file pntd.0005599.s004.docx]

**Supplementary Information**

**ICOSA schistosomiasis survey sample size calculations**

Sample size calculations were performed by the Biostatistician at the Schistosomiasis Control Initiative. Survey data from Uganda (*S. mansoni*) and Burkina Faso (*S. haematobium*) were first used to derive estimates of the intra-cluster correlation coefficient, ICC. The ICC was estimated at 0.33 for *S. mansoni* prevalence across a substantial area of Uganda and at 0.35 for *S. haematobium* prevalence across Burkina Faso. Since ICOSA surveys would estimate prevalence at a district level, and districts are likely to be less ecologically heterogeneous than these large geographic areas, we used an estimated ICC value of 0.2. Sample size calculations were performed to find a two-stage cluster survey design that would be capable of estimating 50% prevalence with a 10 percentage point margin of error on the 95% confidence interval, using calculations from Lohr 2009. This led to a recommended sample size of 20 schools per district, and 30 children sampled per school.

**Figure S1**: Comparison of true primary school locations (A and B) with those estimated using population-based approximations (C and D) in Malawi and Kenya respectively.


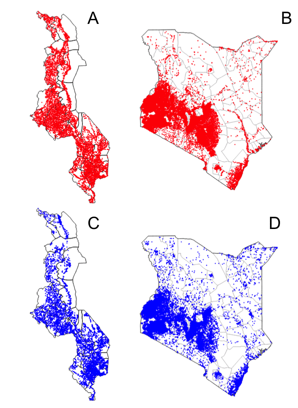


**Figure S2**: Correlation between the number of real primary schools and the number of primary school locations simulated using a population-based approximation across 0.3 decimal degree grid squares in (A) Malawi and (B) Kenya.


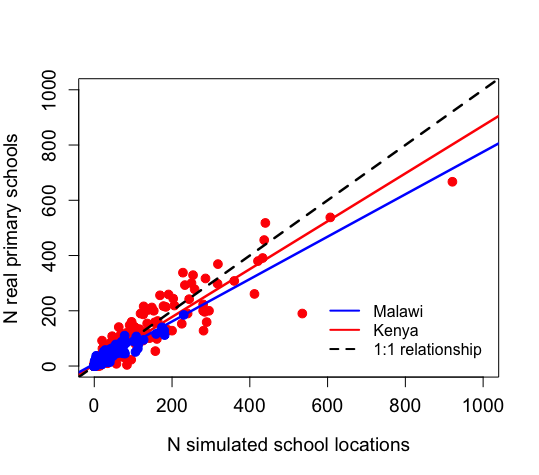


**Figure S3:** Histograms illustrating variation in A) schistosomiasis prevalence across schools and B) district level intra-class correlation coefficients (ICC) for schistosomiasis prevalence, in empirical mapping datasets from Cote d’Ivoire, Malawi and Liberia.

**
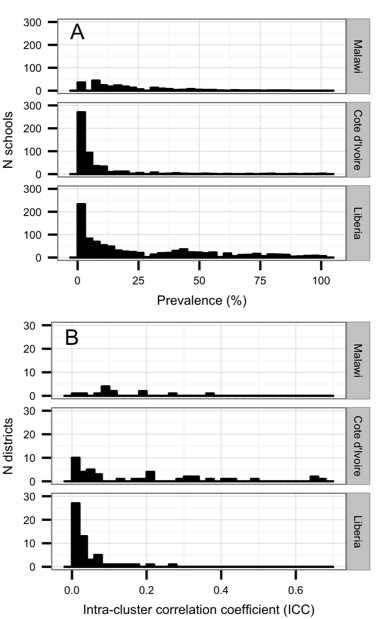
**

**Figure S4:** Violin plots illustrating the range of school prevalence values for schistosomiasis (any species) in each district within baseline mapping data. Black diamonds indicate the district prevalence estimate, and horizontal dashed lines mark the WHO endemicity/treatment thresholds for schistosomiasis PCT (1-10% low endemicity, 10-50% moderate endemicity, 50% and above high endemicity). Wide variation among schools within a district of a given endemicity class is evident.

**
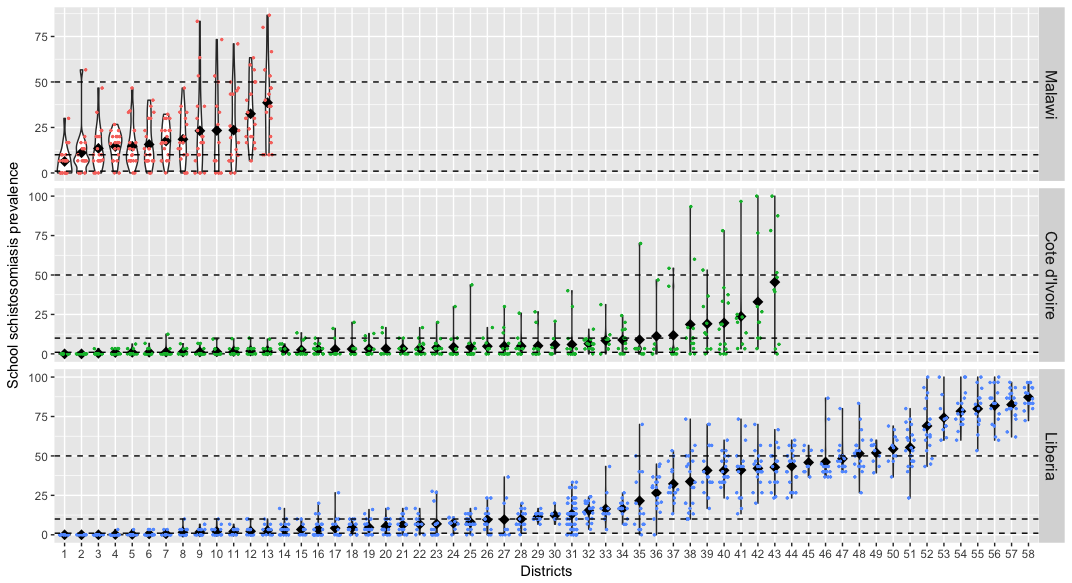
**

**Figure S5:** The effect of survey design on the accuracy of district-level schistosomiasis treatment decisions, using a gold standard simulated dataset of 143 districts from Malawi, Côte d’Ivoire and Liberia. A) the proportion of times a survey failed to detect endemic schistosomiasis, B) the proportion of times districts were wrongly classified into either a higher or lower treatment class, and C) the proportion of times districts were classified into a treatment class below their true class.


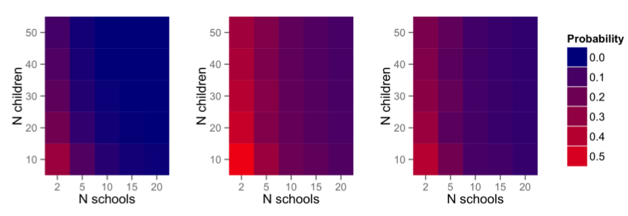


**Figure S6**: The effect of mapping survey design on the accuracy of schistosomiasis treatment decisions for a given total sample size per district from simulations. (A) the proportion of times surveys failed to detect endemic schistosomiasis in districts (B) precision of district-level prevalence estimates, reflected by the mean width of exact 95% confidence intervals (C) the proportion of times districts were classified into the wrong endemicity category (D) the proportion of times districts were classified into an endemicity category below their true class.


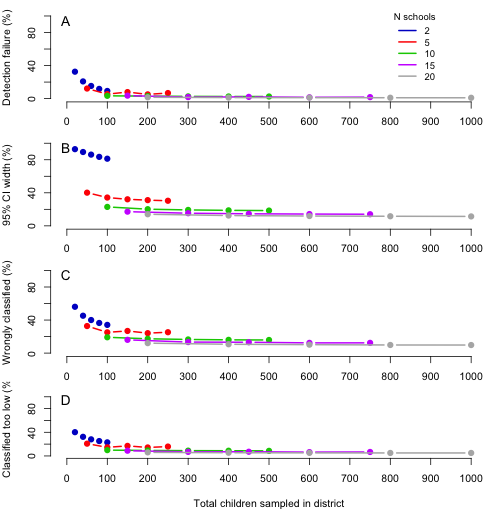


**Figure S7**: The effect of alternative assignment rules on the probability of classifying districts into schistosomiasis endemicity classes below than their true class. In all graphs, the number of children sampled per school was kept constant at 30. The following four rules were considered for using prevalence estimates to classify districts (A) classify using the unadjusted point prevalence estimate (B) boosting districts where the point estimate is within 2 percentage points of the moderate or high endemicity class thresholds (C) boosting districts where the point estimate is within 5 percentage points of the moderate or high endemicity class thresholds (D) classify into a higher endemicity class if the upper 95% confidence limit overlaps that threshold.


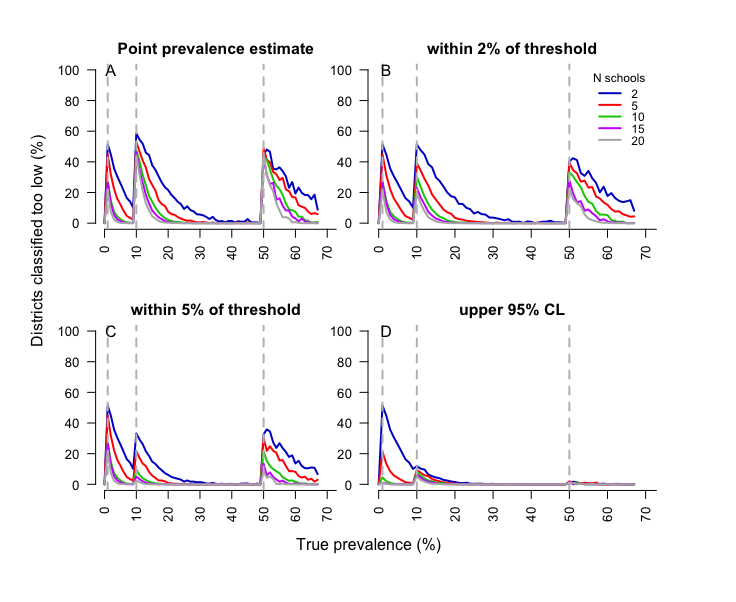


**Figure S8:** Relationship between four measures of survey accuracy from simulations, and district size. Districts are grouped into size tertiles, small (<1438km^2^), medium (1439-4394km^2^) and large (>4395km^2^). Data are plotted for surveys involving 30 children sampled per school.

**
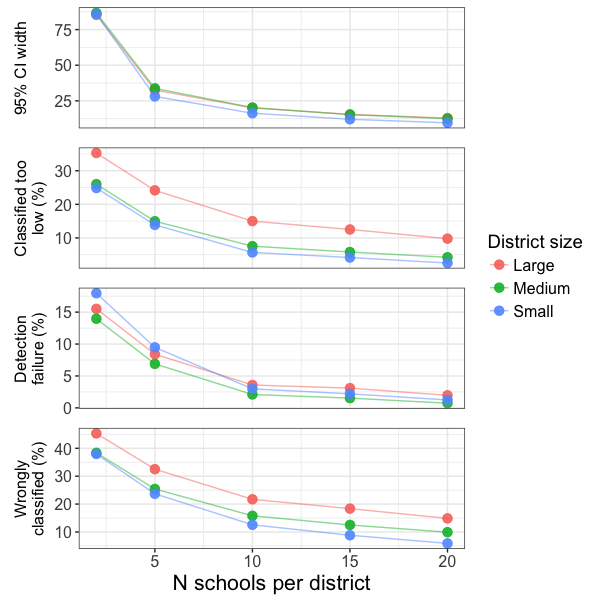
**

**Figure S9**: Simulation results illustrating the proportion of times schools would have been (A) wrongly classified or (B) classified into an endemicity class below their true class, according to school-level prevalence under varying survey design.

**
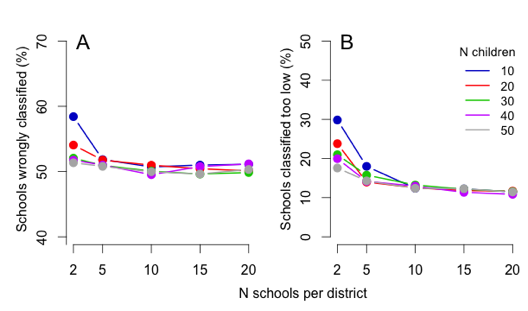
**

**Figure S10**: Sensitivity (percentage change) of total survey cost to changes in three key cost drivers: Capital item lifespan (±20%), survey staff salaries (±20%) and the number of teams simultaneously performing the survey (1 to 5).


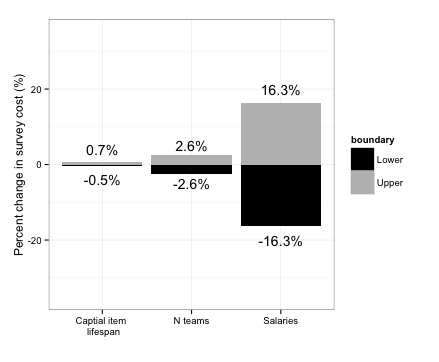


**Figure S11:** Effect of the number of schools surveyed per district, and the number of children sampled at each school, on the estimated cost of a mapping survey in Malawi.

**
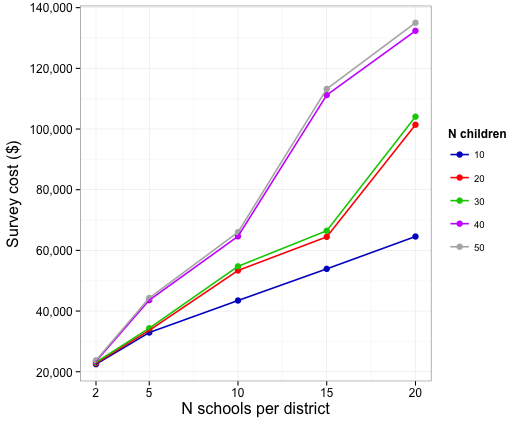
**

**Figure S12:** Variation in survey team salary had almost no effect on the relative cost efficiency of alternate survey designs in Malawi. Line colour represents district classification rules (blue: point prevalence estimate; red: point prevalence with 2 percentage point boost at thresholds; green: point prevalence with 5 percentage point boost at thresholds; black: upper 95% confidence limit).


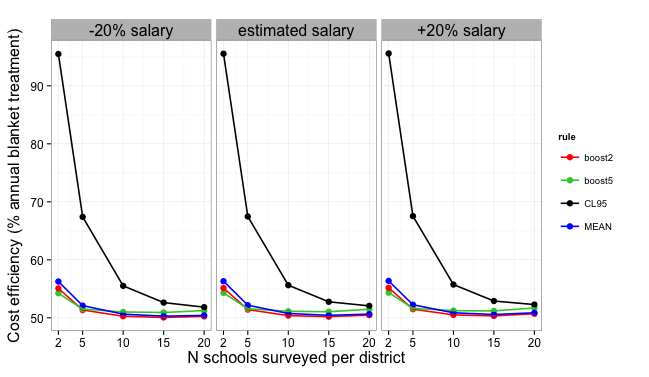


**Table S1:** Country data used in generation of gold standard datasets. Only districts in which 20 or more school locations were assigned were included in simulations, such that all sampling strategies could be compared across all countries.

| **Country** | **N primary schools** | **Average SAC per school** | **N districts simulated**  **(N districts in country)** | **Data sources** |
| --- | --- | --- | --- | --- |
| Malawi | 5,239 | 400 | 26 (26) | ^1,2^ Fully geo-referenced primary school database ^3^ DIVA-GIS website |
| Côte d’Ivoire | 11,429 | 250 | 78 (82) | ^1^ Primary school list obtained from the Ministry of Health; ^2^ Enrolment figures collected in 8 districts during ICOSA mapping; ^3^ Heath district shapefile obtained from Ministry of Health |
| Liberia | 2,785 | 150 | 39 (66) | ^1, 2^ 2013 EMIS National Statistical Booklet; ^3^ DIVA-GIS website |

^1^ Source for number of primary schools nationwide

^2^ Source for average SAC per school

^3^ Source for district number and geographic boundaries

**Table S2:** Assumed useful lifespan of capital equipment used in calculations of mapping survey costs.

| **Capital equipment* (Fixed)** | **Useful lifespan (years)** |
| --- | --- |
| Basins | 5 |
| Brushes | 2 |
| Buckets | 5 |
| Droppers | 2 |
| Forceps | 5 |
| GPS | 5 |
| Hole punch | 5 |
| Microscope | 5 |
| Sieving mesh | 3 |
| Slide boxes | 5 |
| Tally counters | 5 |
| **Capital equipment* (Variable)** |  |
| Filter Holders | 4 |
| Stool pots | 5 |
| Syringes | 5 |
| Kato-Katz kits (template and plastic spatula) | 4 |
| Urine Pots | 5 |

**Table S3:** Estimated number of days required to survey a district under different mapping survey designs, based on field experience in Malawi. Survey duration estimates included a day for traveling to/from the field and account for the fact that school visits cannot occur on weekends.

|  |  | **Number of schools** | | | | |
| --- | --- | --- | --- | --- | --- | --- |
|  |  | **2** | **5** | **10** | **15** | **20** |
| **Number of children tested per school** | **10** | 2.1 | 3.1 | 4.1 | 5.1 | 6.1 |
|  | **20** | 2.1 | 3.1 | 5.1 | 6.1 | 10.1 |
|  | **30** | 2.1 | 3.1 | 5.1 | 6.1 | 10.1 |
|  | **40** | 2.1 | 4.1 | 6.1 | 11.1 | 13.1 |
|  | **50** | 2.1 | 4.1 | 6.1 | 11.1 | 13.1 |

# Table S4: The mean proportion of simulated surveys failing to detect treatable levels (≥1%) of schistosomiasis in simulated datasets, according to district-level prevalence classes (low: 1-10%; moderate: 10-50%, high: 50% and above).

|  |  | **Surveys failing to detect ≥1% schistosomiasis prevalence (%)** | | |
| --- | --- | --- | --- | --- |
| **N schools** | **N children** | ***Low*** | ***Moderate*** | ***High*** |
| 2 | 10 | 52.88 | 11.91 | 0.02 |
| 2 | 20 | 36.14 | 4.78 | 0.00 |
| 2 | 30 | 27.53 | 2.43 | 0.00 |
| 2 | 40 | 21.61 | 1.30 | 0.00 |
| 2 | 50 | 17.16 | 0.83 | 0.00 |
| 5 | 10 | 22.58 | 1.15 | 0.00 |
| 5 | 20 | 10.30 | 0.18 | 0.00 |
| 5 | 30 | 5.81 | 0.05 | 0.00 |
| 5 | 40 | 3.33 | 0.02 | 0.00 |
| 5 | 50 | 2.07 | 0.00 | 0.00 |
| 10 | 10 | 6.48 | 0.06 | 0.00 |
| 10 | 20 | 1.77 | 0.00 | 0.00 |
| 10 | 30 | 0.65 | 0.00 | 0.00 |
| 10 | 40 | 0.30 | 0.00 | 0.00 |
| 10 | 50 | 0.12 | 0.00 | 0.00 |
| 15 | 10 | 2.12 | 0.00 | 0.00 |
| 15 | 20 | 0.38 | 0.00 | 0.00 |
| 15 | 30 | 0.11 | 0.00 | 0.00 |
| 15 | 40 | 0.03 | 0.00 | 0.00 |
| 15 | 50 | 0.01 | 0.00 | 0.00 |
| 20 | 10 | 0.82 | 0.00 | 0.00 |
| 20 | 20 | 0.11 | 0.00 | 0.00 |
| 20 | 30 | 0.02 | 0.00 | 0.00 |
| 20 | 40 | 0.00 | 0.00 | 0.00 |
| 20 | 50 | 0.00 | 0.00 | 0.00 |

**Table S5:** The proportion (%) of times districts were classified in a treatment class below their true class, according to survey design and the rule used to assign districts to classes.

| **N schools** | **N children** | **Point estimate** | **2% boost** | **5% boost** | **95% confidence limit** |
| --- | --- | --- | --- | --- | --- |
| 2 | 10 | 40.19 | 40.19 | 33.35 | 32.62 |
| 2 | 20 | 32.33 | 32.33 | 24.94 | 20.95 |
| 2 | 30 | 28.07 | 25.57 | 20.48 | 15.99 |
| 2 | 40 | 25.15 | 23.35 | 17.51 | 12.64 |
| 2 | 50 | 22.99 | 19.99 | 15.19 | 10.23 |
| 5 | 10 | 20.77 | 18.07 | 15.65 | 12.20 |
| 5 | 20 | 14.56 | 11.65 | 8.05 | 6.43 |
| 5 | 30 | 17.02 | 14.04 | 10.77 | 4.15 |
| 5 | 40 | 14.35 | 11.34 | 7.69 | 2.97 |
| 5 | 50 | 15.85 | 12.81 | 9.40 | 2.45 |
| 10 | 10 | 9.85 | 7.17 | 4.60 | 3.88 |
| 10 | 20 | 9.31 | 6.60 | 4.04 | 1.57 |
| 10 | 30 | 8.98 | 6.22 | 3.64 | 1.09 |
| 10 | 40 | 8.83 | 6.06 | 3.54 | 0.96 |
| 10 | 50 | 8.61 | 5.83 | 3.44 | 0.90 |
| 15 | 10 | 8.86 | 6.25 | 4.27 | 1.45 |
| 15 | 20 | 6.89 | 4.20 | 2.27 | 0.65 |
| 15 | 30 | 7.10 | 4.39 | 2.54 | 0.58 |
| 15 | 40 | 6.50 | 3.85 | 2.00 | 0.59 |
| 15 | 50 | 6.72 | 4.00 | 2.20 | 0.59 |
| 20 | 10 | 6.07 | 3.50 | 1.90 | 0.68 |
| 20 | 20 | 5.46 | 2.94 | 1.45 | 0.38 |
| 20 | 30 | 5.22 | 2.77 | 1.35 | 0.39 |
| 20 | 40 | 5.07 | 2.57 | 1.25 | 0.43 |
| 20 | 50 | 5.06 | 2.50 | 1.17 | 0.44 |

**Table S6:** Estimated cost (in $ USD) of different surveys in Malawi covering all 26 districts, according to the number of schools visited per district and the number of children sampled per school.

|  |  | **Number of schools** | | | | |
| --- | --- | --- | --- | --- | --- | --- |
|  |  | **2** | **5** | **10** | **15** | **20** |
| **Number of children tested per school** | **10** | 22,482 | 32,916 | 43,468 | 53,896 | 64,571 |
|  | **20** | 22,800 | 33,626 | 53,372 | 64,452 | 101,409 |
|  | **30** | 23,118 | 34,335 | 54,734 | 66,465 | 104,074 |
|  | **40** | 23,437 | 43,588 | 64,638 | 111,194 | 132,368 |
|  | **50** | 23,755 | 44,297 | 65,999 | 113,207 | 135,033 |

**References**

Lohr, S. 2009 *Sampling: design and analysis*. Cengage Learning.
